# Supplementary material for: Palladium-Catalyzed Direct (Het)arylation Reactions of Benzo[1,2-d:4,5-d′]bis([1,2,3]thiadiazole and 4,8-Dibromobenzo[1,2-d:4,5-d′]bis([1,2,3]thiadiazole)
Source: Molecules. 2023 May 8;28(9):3977. doi: 10.3390/molecules28093977 (PMC10180130; doi:10.3390/molecules28093977)

Supplementary Materials

Palladium-catalyzed direct (het)arylation reactions of benzo[1,2-*d*:4,5-*d*']bis([1,2,3]thiadiazole and 4,8-dibromobenzo[1,2-*d*:4,5-*d*']bis([1,2,3]thiadiazole)

Timofey N. Chmovzh[1,2], Timofey A. Kudryashev[1,3], Daria A. Alekhina[1,4] and Oleg A. Rakitin[1]*

[1] *N. D. Zelinsky Institute of Organic Chemistry, Russian Academy of Sciences, 119991 Moscow, Russian Federation; orakitin@ioc.ac.ru (O.A.R.); tim1661@yandex.ru (T.N.C.);*

[2] *Nanotechnology Education and Research Center, South Ural State University, 454080 Chelyabinsk, Russia; chmovzhtn@susu.ru (T.N.C.);*

[3] *Department of Chemistry, Moscow State University, 119899 Moscow, Russia*

[4] *Higher Chemical College, Mendeleev University of Chemical Technology of Russia, Miusskaya Sq. 9, 125047 Moscow, Russia*

* *Correspondence: orakitin@ioc.ac.ru; Tel.: +7 499 135 5327*

Table of contents:

^{1}H and ^{13}C NMR spectra……………………………………………………………......S2

4-Bromo-8-(5-(2-ethylhexyl)thiophen-2-yl)benzo[1,2-d:4,5-d']bis([1,2,3]thiadiazole) (4a)

^{1}H NMR (300 MHz)

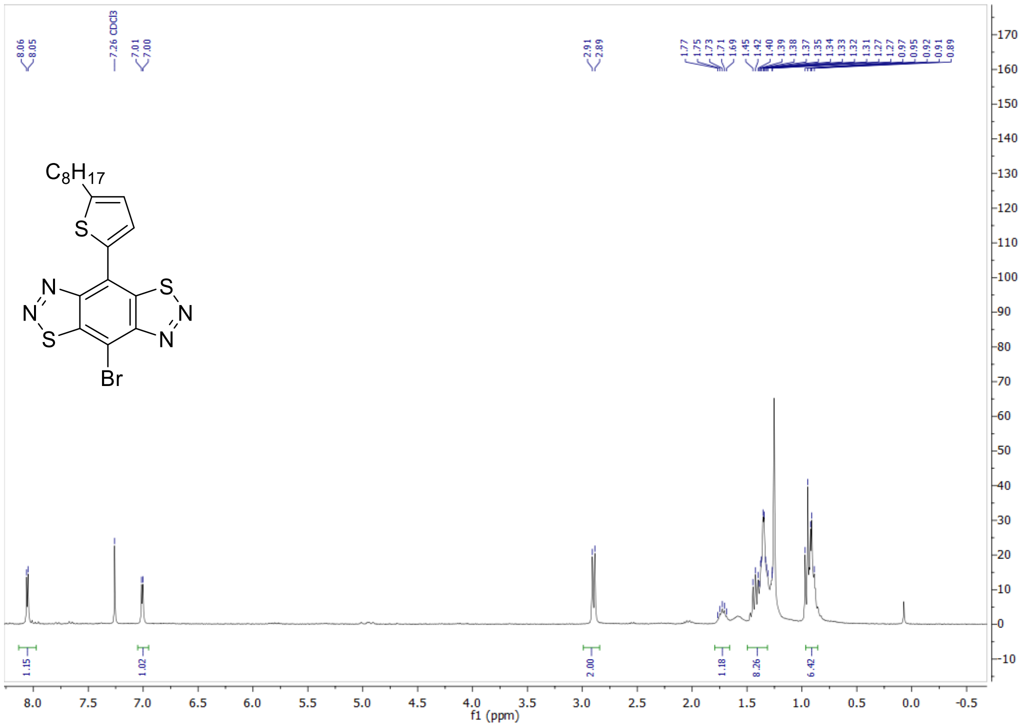

^{13}C NMR(75 MHz)

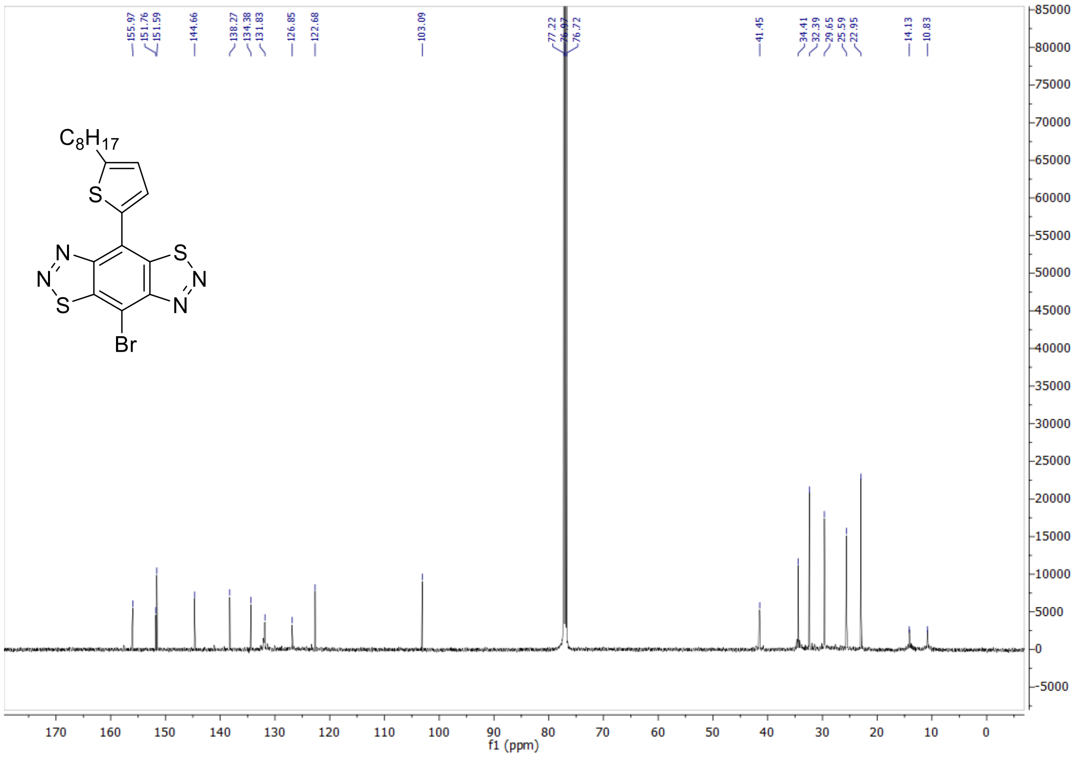

4-Bromo-8-(thiophen-2-yl)benzo[1,2-d:4,5-d']bis([1,2,3]thiadiazole) (4b)

^{1}H NMR (300 MHz)

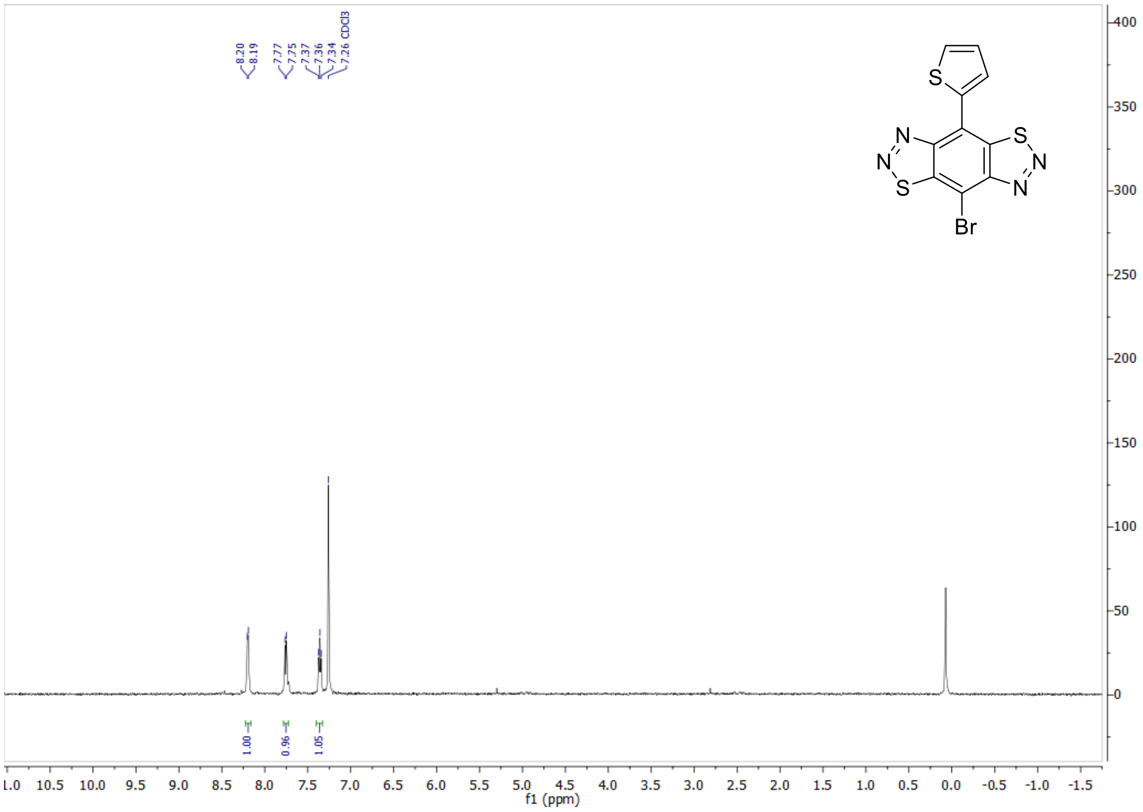

^{13}C NMR(75 MHz)

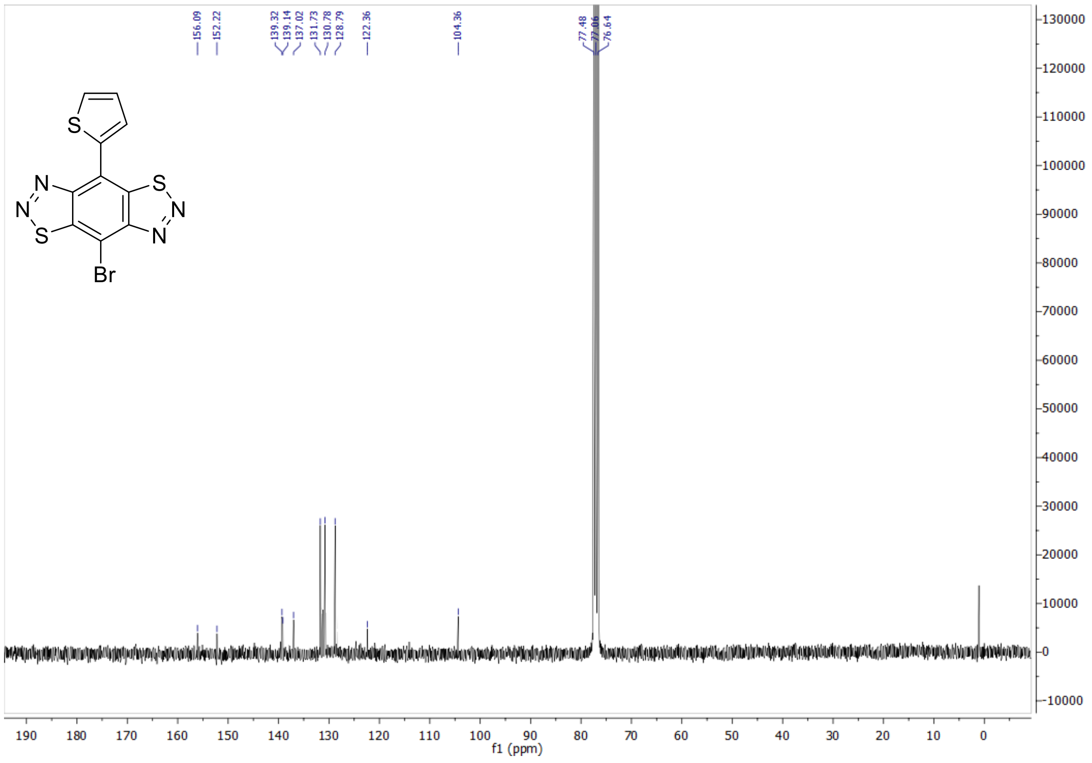

4-Bromo-8-(4-hexylthiophen-2-yl)benzo[1,2-d:4,5-d']bis([1,2,3]thiadiazole) (4c)

^{1}H NMR (300 MHz)

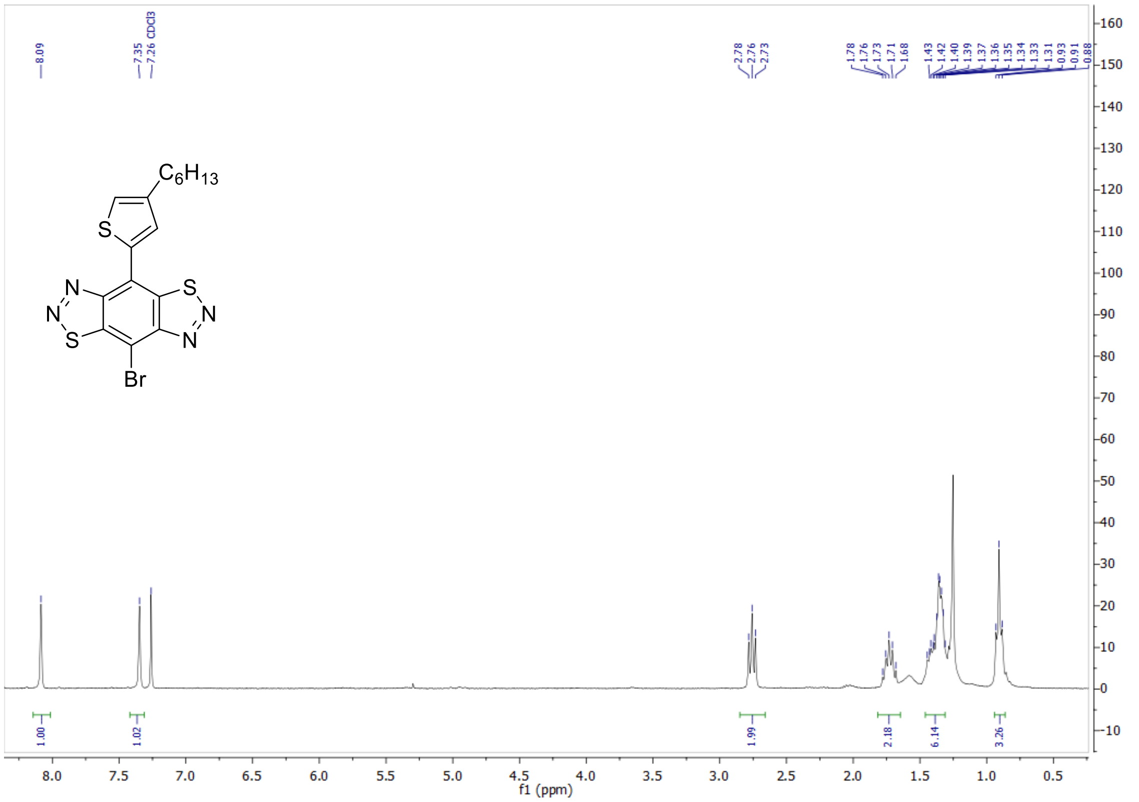

^{13}C NMR(75 MHz)

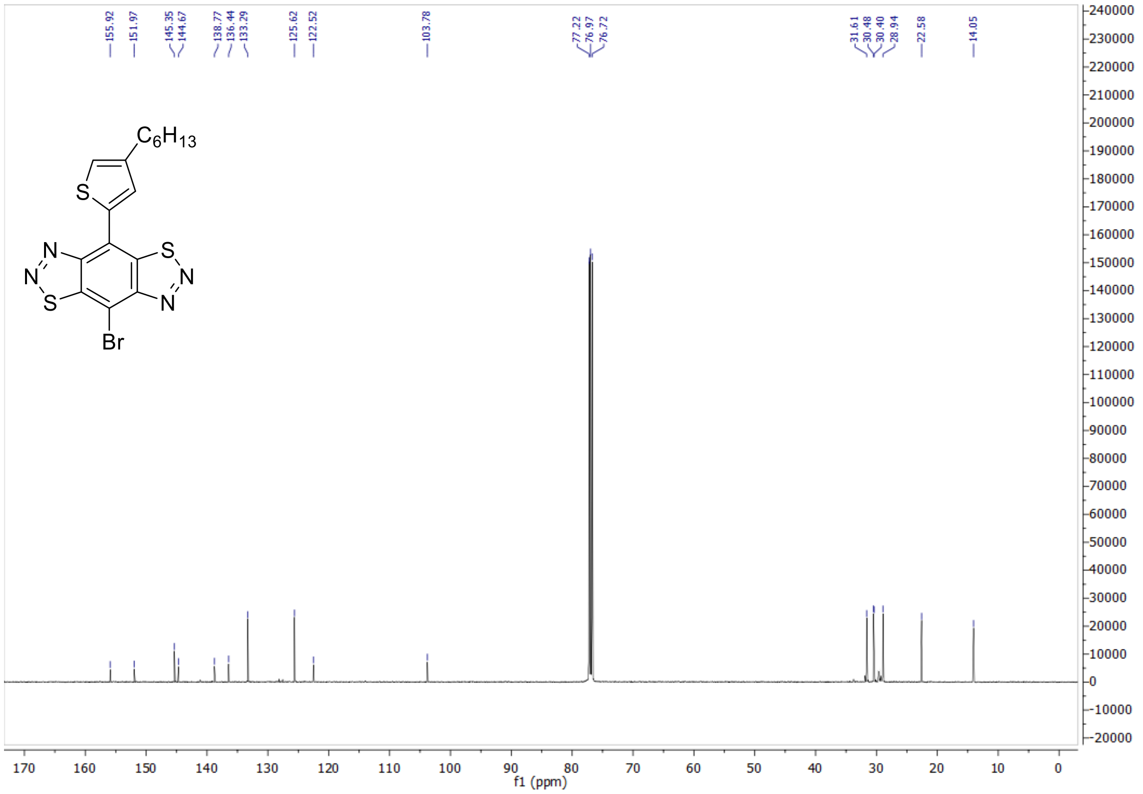

4-([2,2'-Bithiophen]-5-yl)-8-bromobenzo[1,2-d:4,5-d']bis([1,2,3]thiadiazole) (4d)

^{1}H NMR (300 MHz)

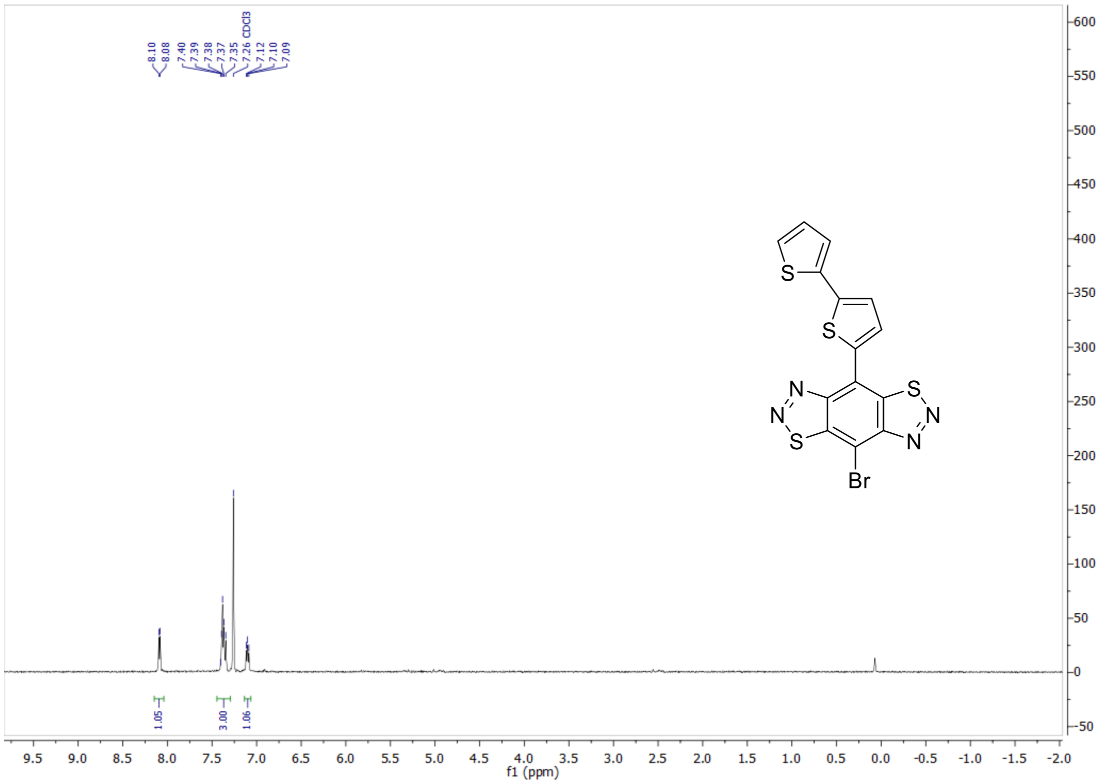

^{13}C NMR(75 MHz)

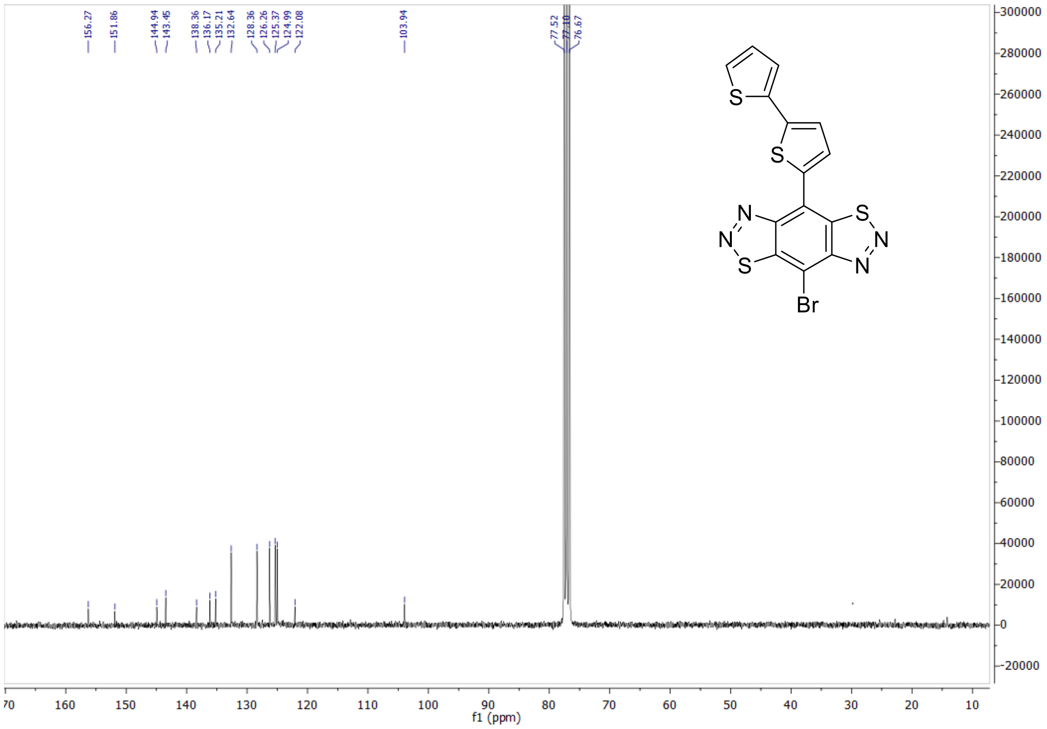

4,8-Bis(4-hexylthiophen-2-yl)benzo[1,2-d:4,5-d']bis([1,2,3]thiadiazole) (5a)

^{1}H NMR (300 MHz)

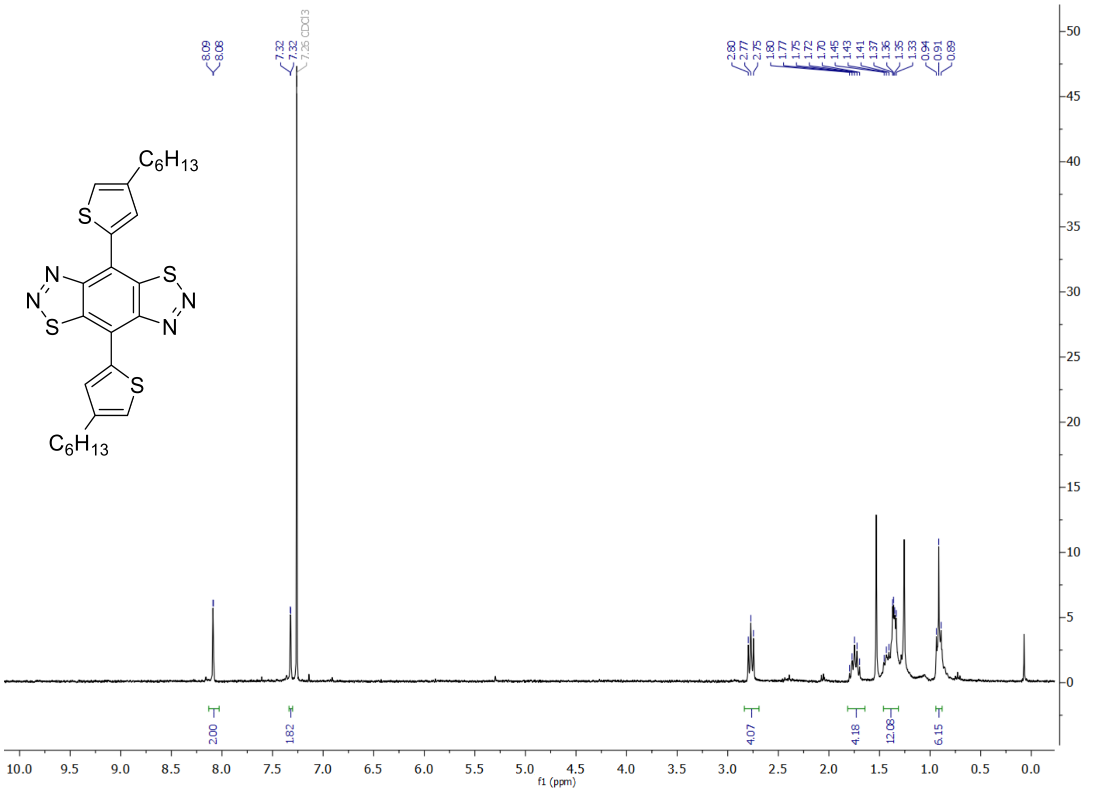

^{13}C NMR(75 MHz)

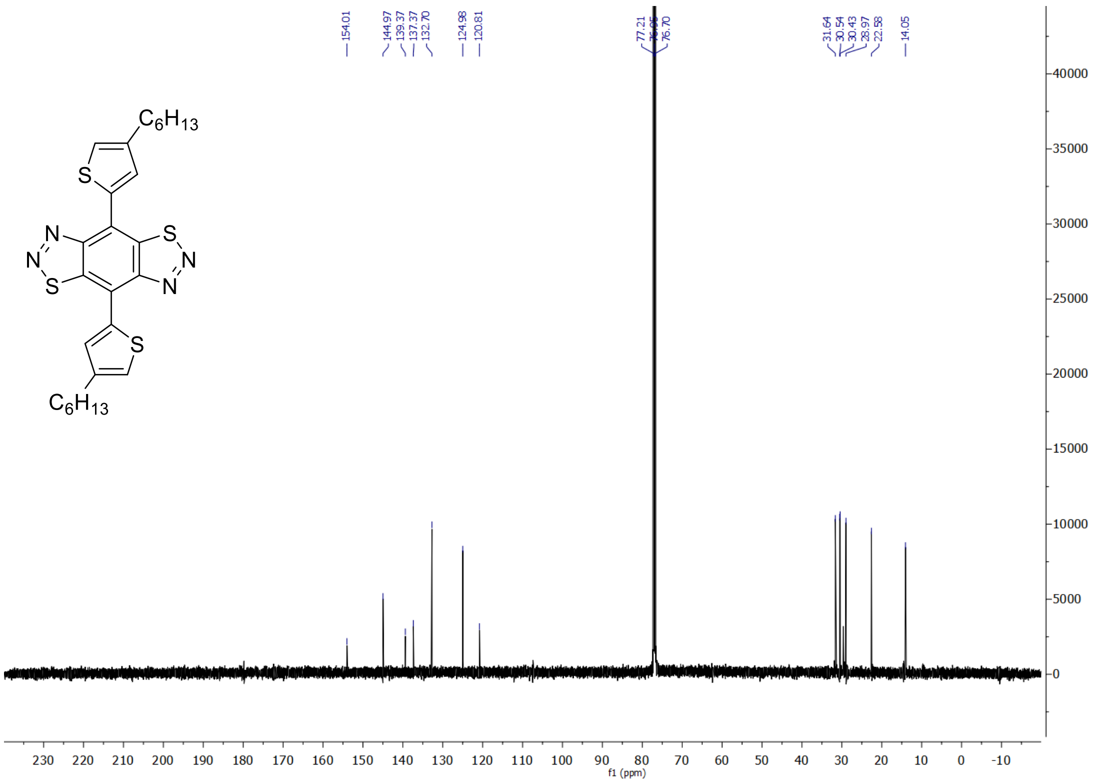

4,8-Di(thiophen-2-yl)benzo[1,2-d:4,5-d']bis([1,2,3]thiadiazole) (5b)

^{1}H NMR (300 MHz)

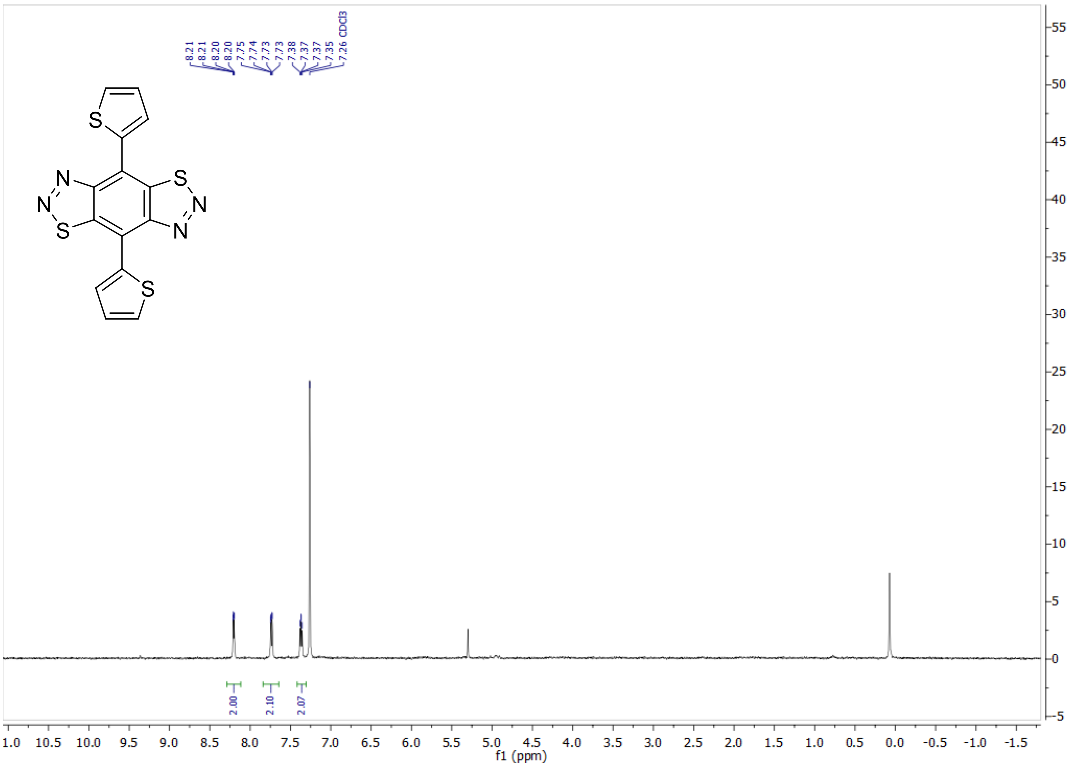

^{13}C NMR(75 MHz)

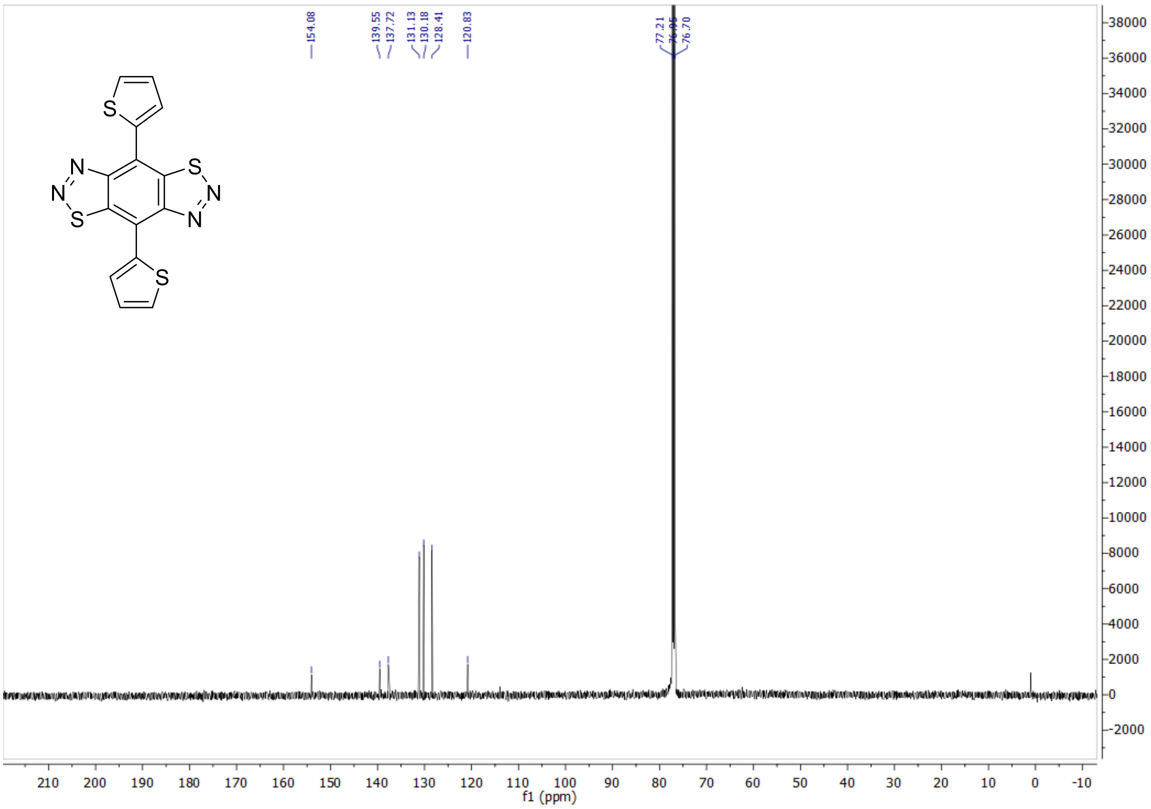

4,8-Bis(4-hexylthiophen-2-yl)benzo[1,2-d:4,5-d']bis([1,2,3]thiadiazole) (5c)

^{1}H NMR (300 MHz)

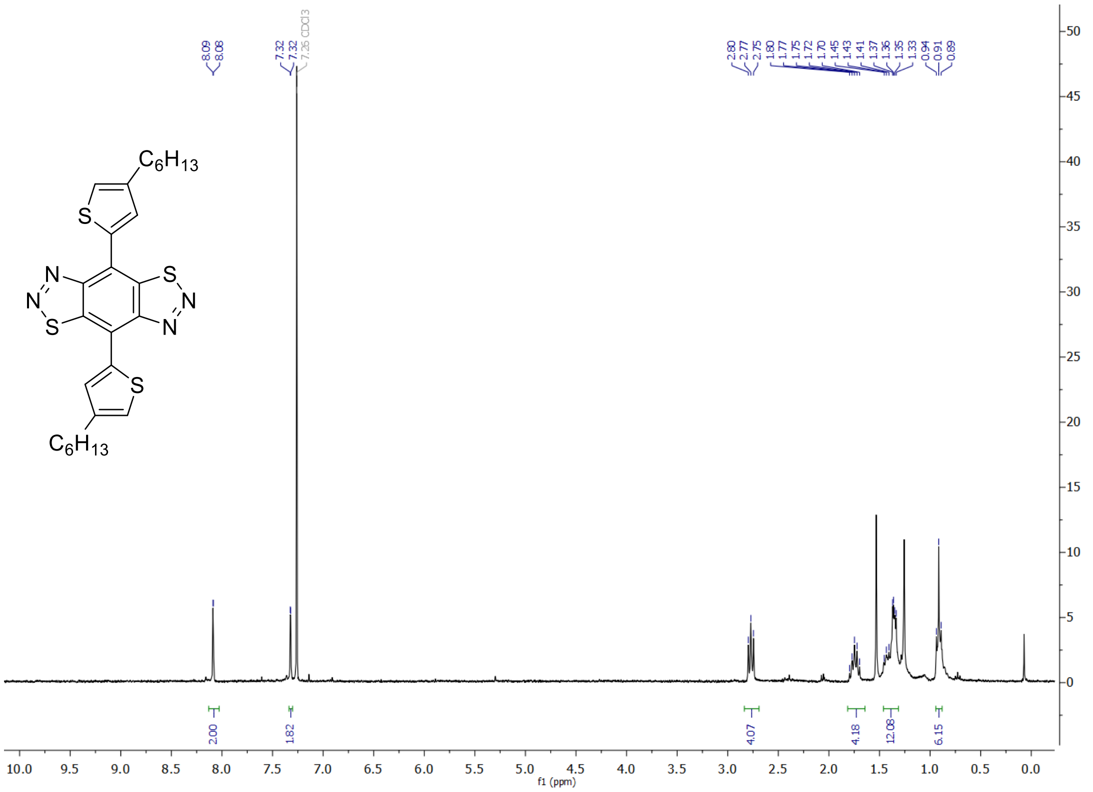

^{13}C NMR(75 MHz)

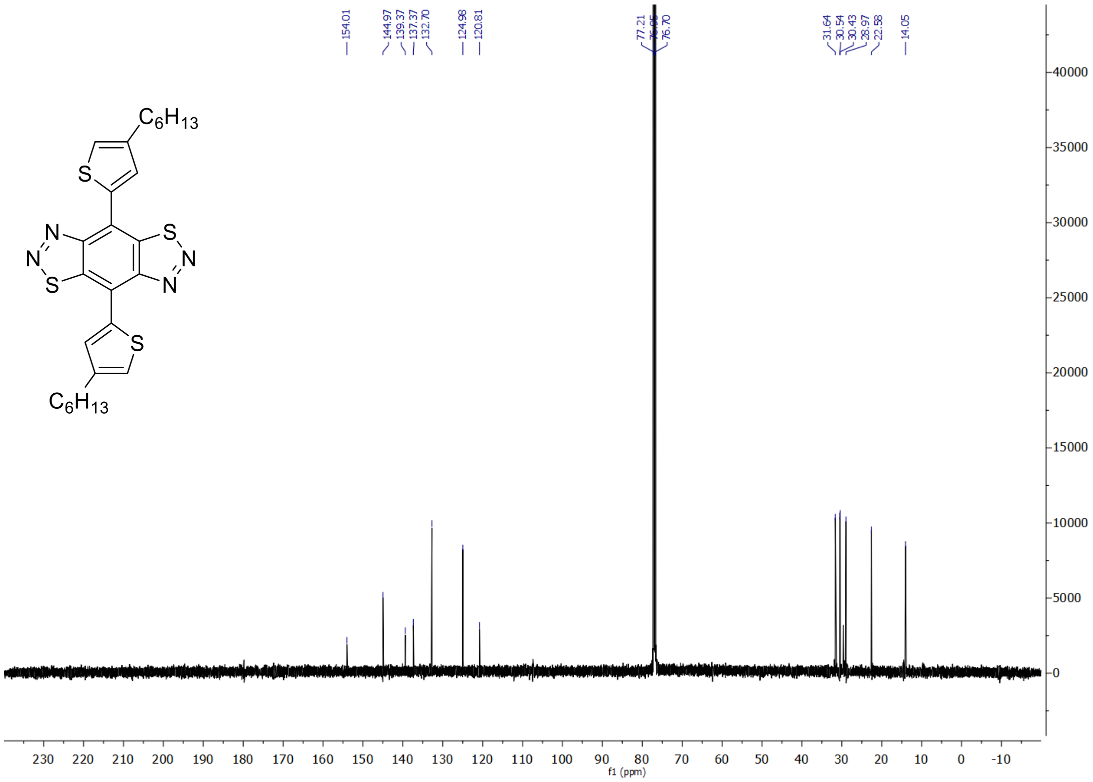

4,8-Di([2,2'-bithiophen]-5-yl)benzo[1,2-d:4,5-d']bis([1,2,3]thiadiazole) (5d)

^{1}H NMR (300 MHz)

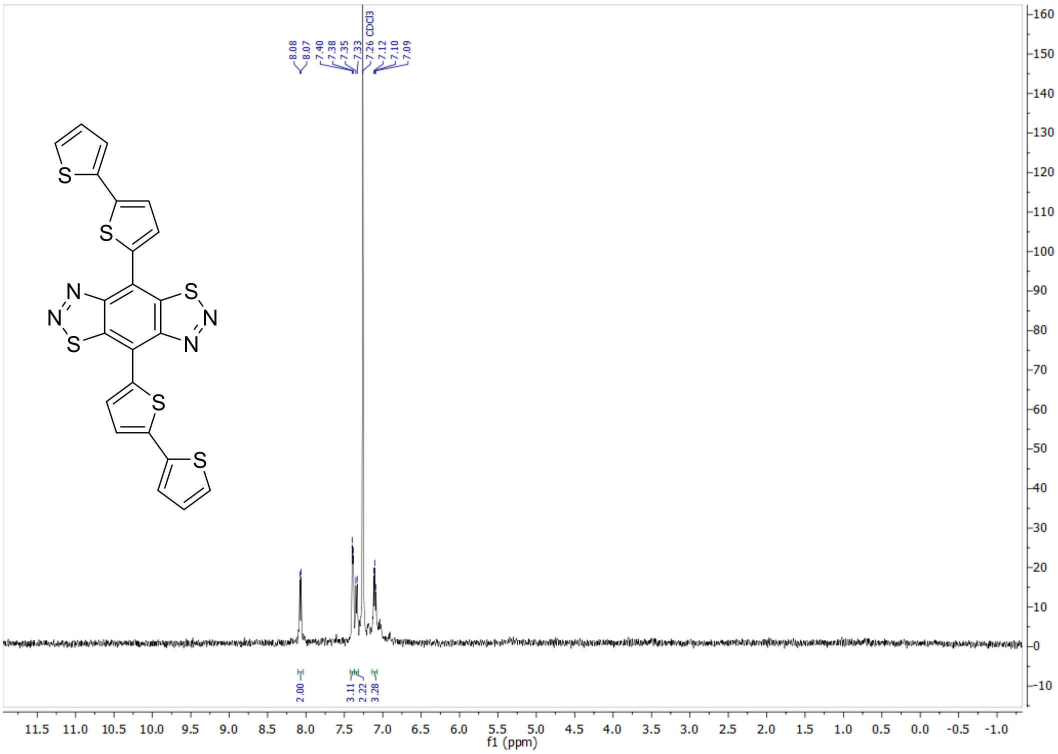

^{13}C NMR(75 MHz)

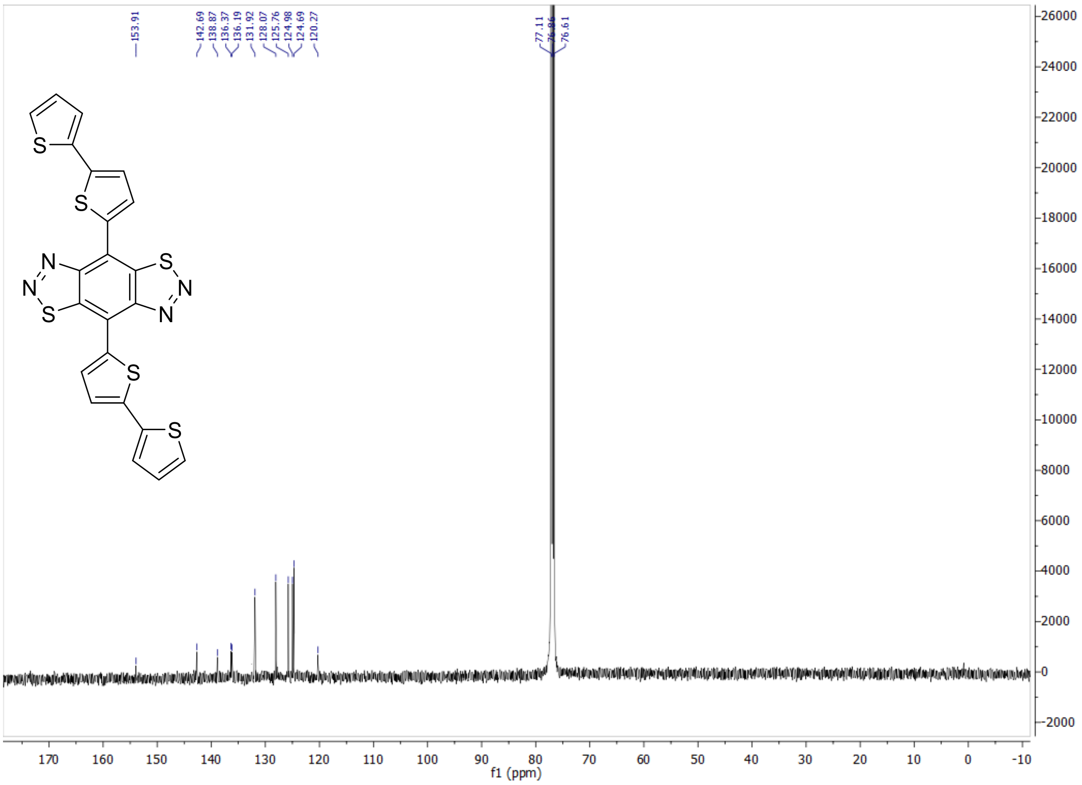

4,8-Bis(5'-(trimethylsilyl)-[2,2'-bithiophen]-5-yl)benzo[1,2-*d*:4,5-*d*']bis([1,2,3]thiadiazole) (5e)

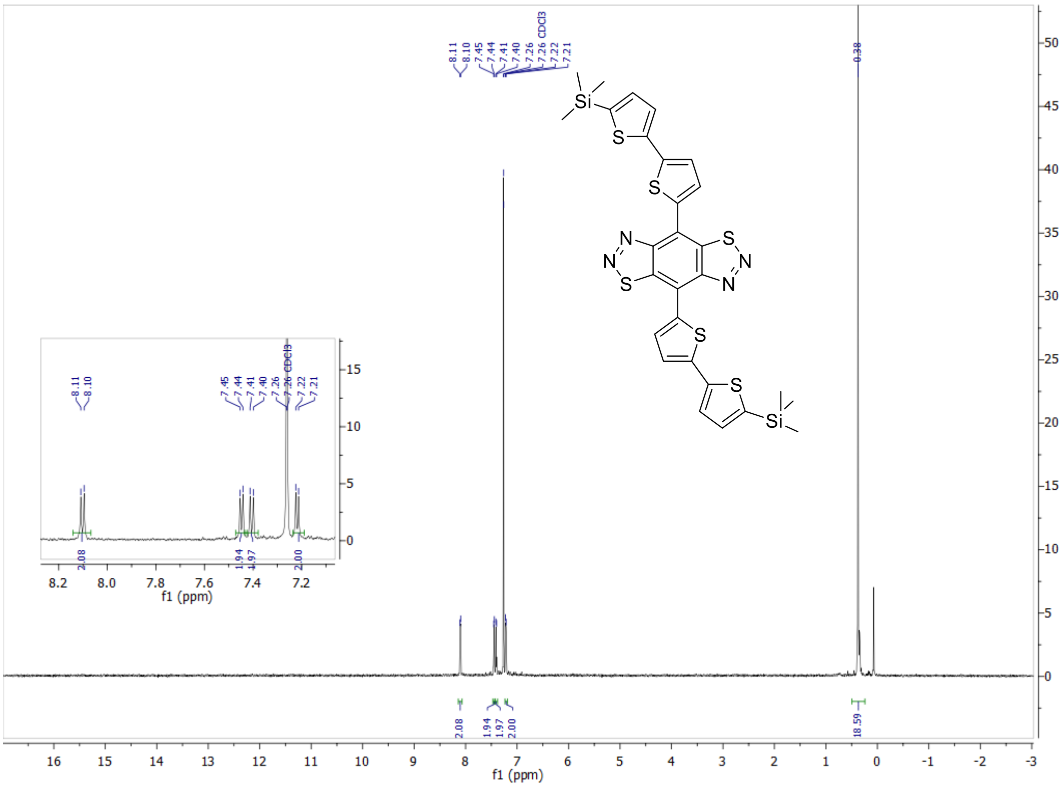

^{13}C NMR(75 MHz)

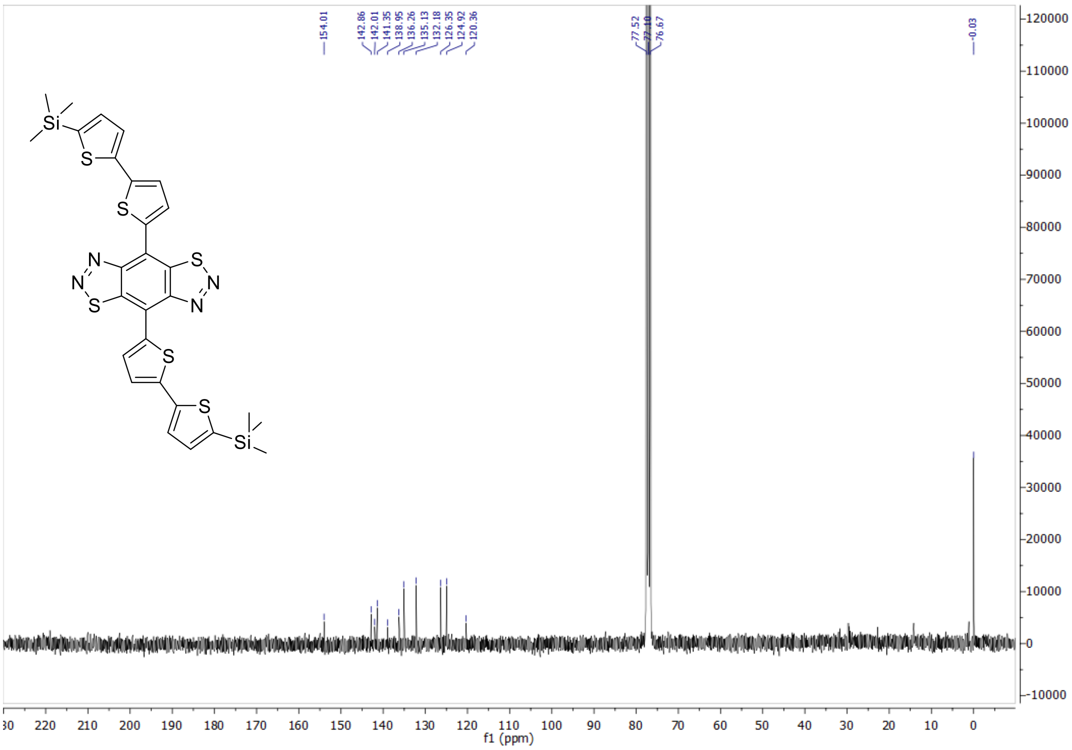

4,8-Diphenylbenzo[1,2-d:4,5-d']bis([1,2,3]thiadiazole) (5f)

^{1}H NMR (300 MHz)

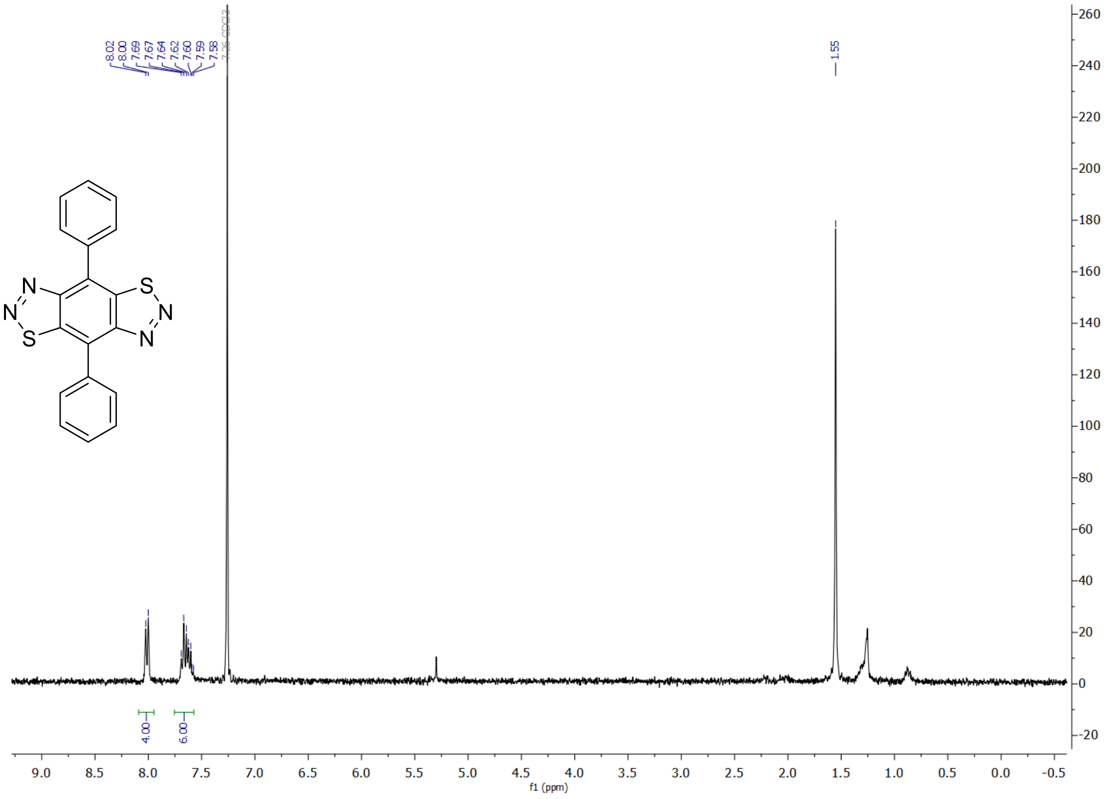

^{13}C NMR(75 MHz)

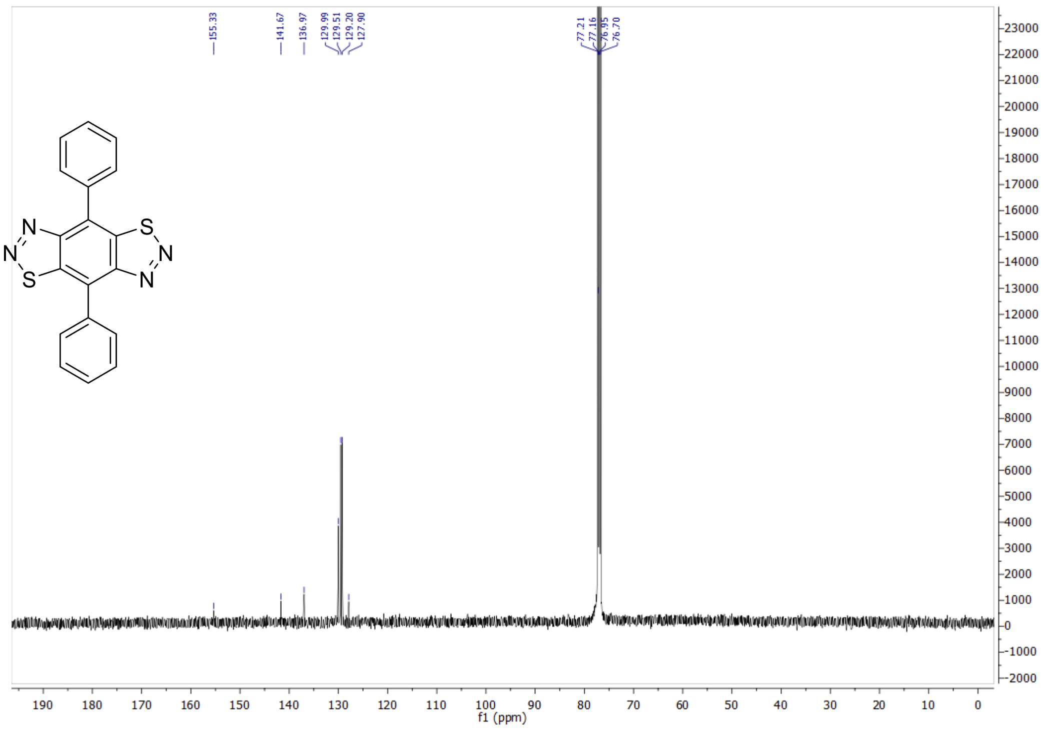

4,8-Di-p-tolylbenzo[1,2-d:4,5-d']bis([1,2,3]thiadiazole) (5g)

^{1}H NMR (300 MHz)

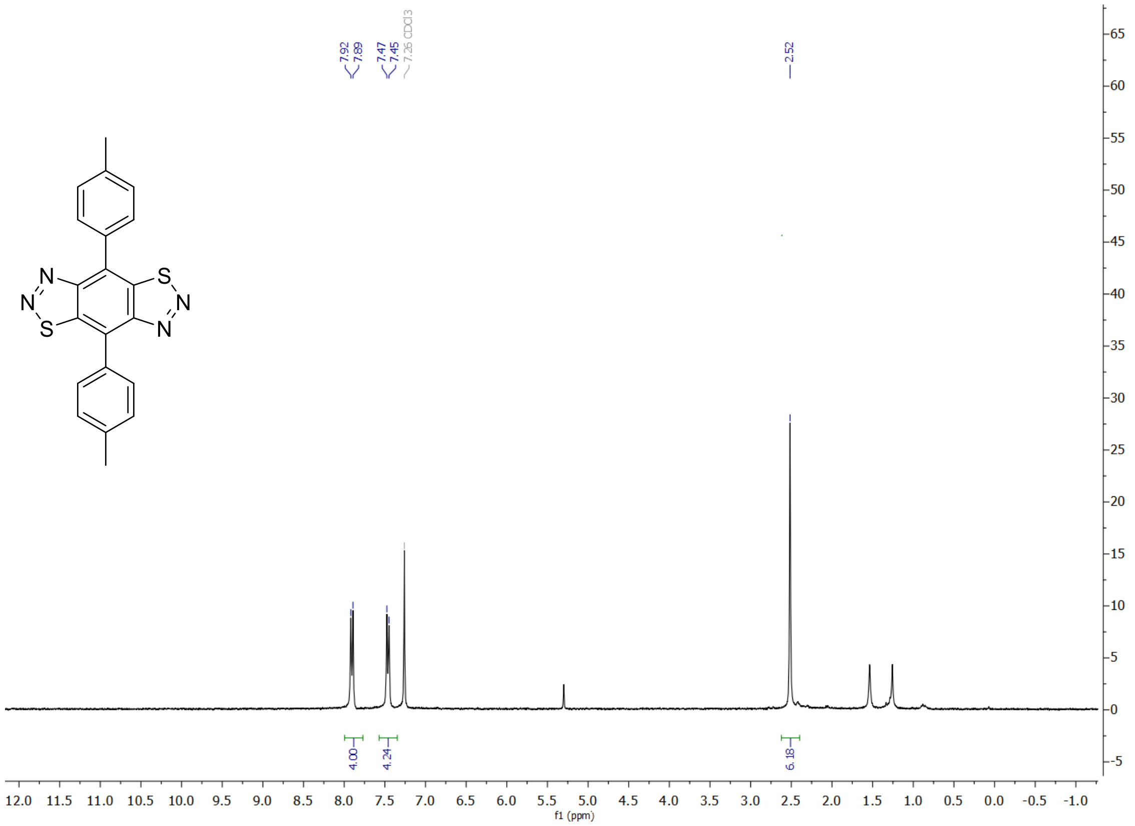

^{13}C NMR(75 MHz)

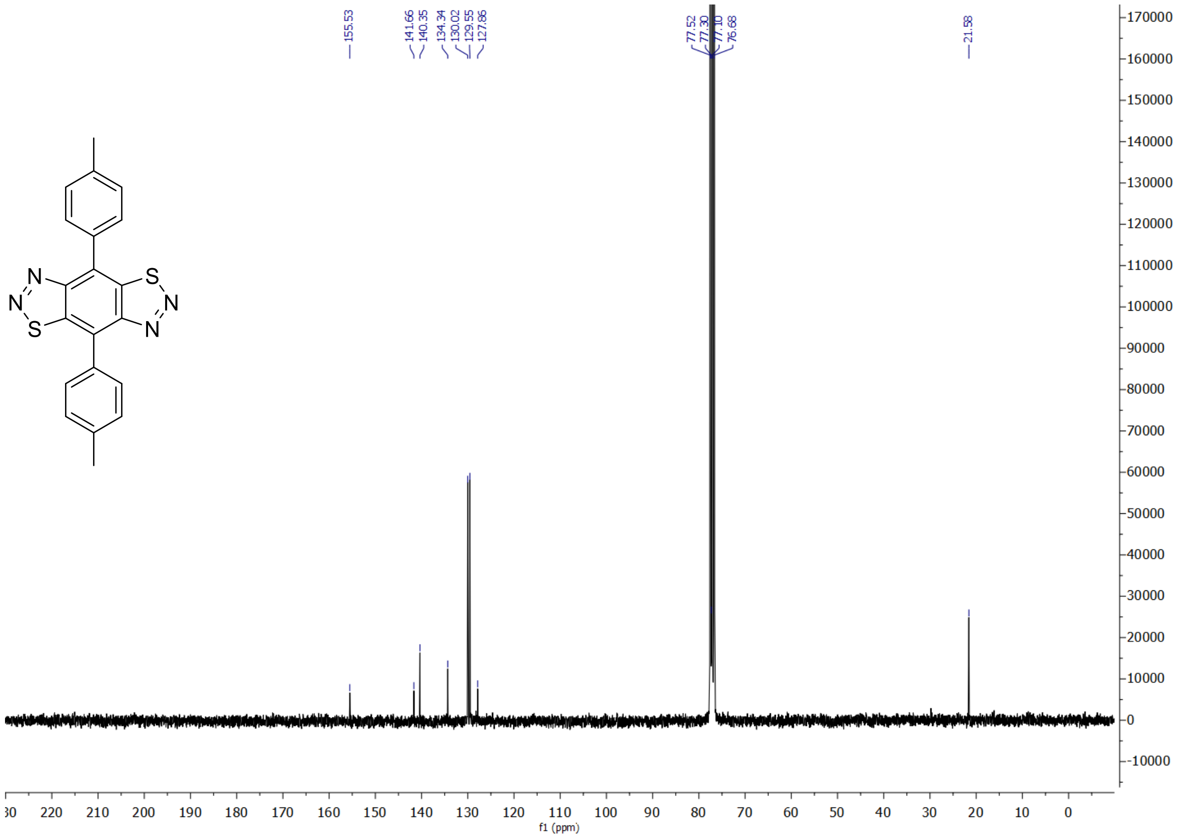

4,8-Bis(4-methoxyphenyl)benzo[1,2-d:4,5-d']bis([1,2,3]thiadiazole) (5h)

^{1}H NMR (300 MHz)

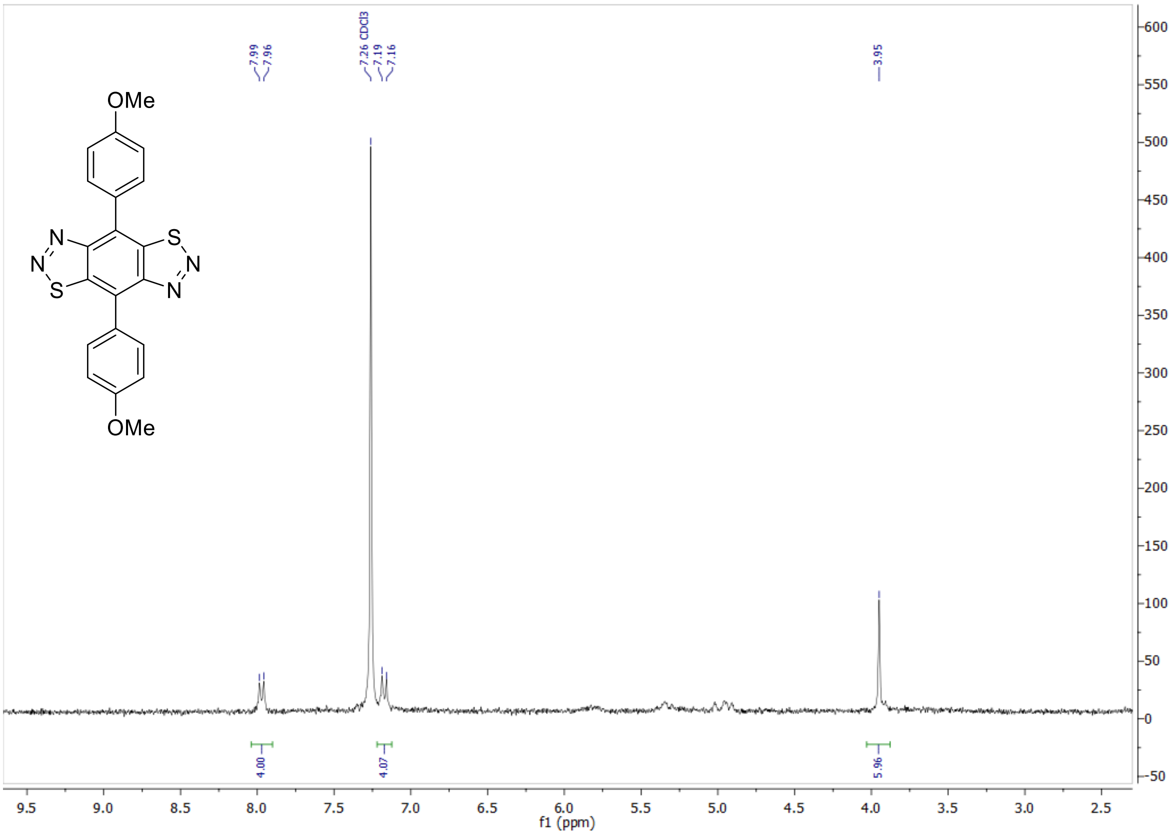

Dimethyl 4,4'-(benzo[1,2-*d*:4,5-*d*']bis([1,2,3]thiadiazole)-4,8-diyl)dibenzoate (5i)

^{1}H NMR (300 MHz)

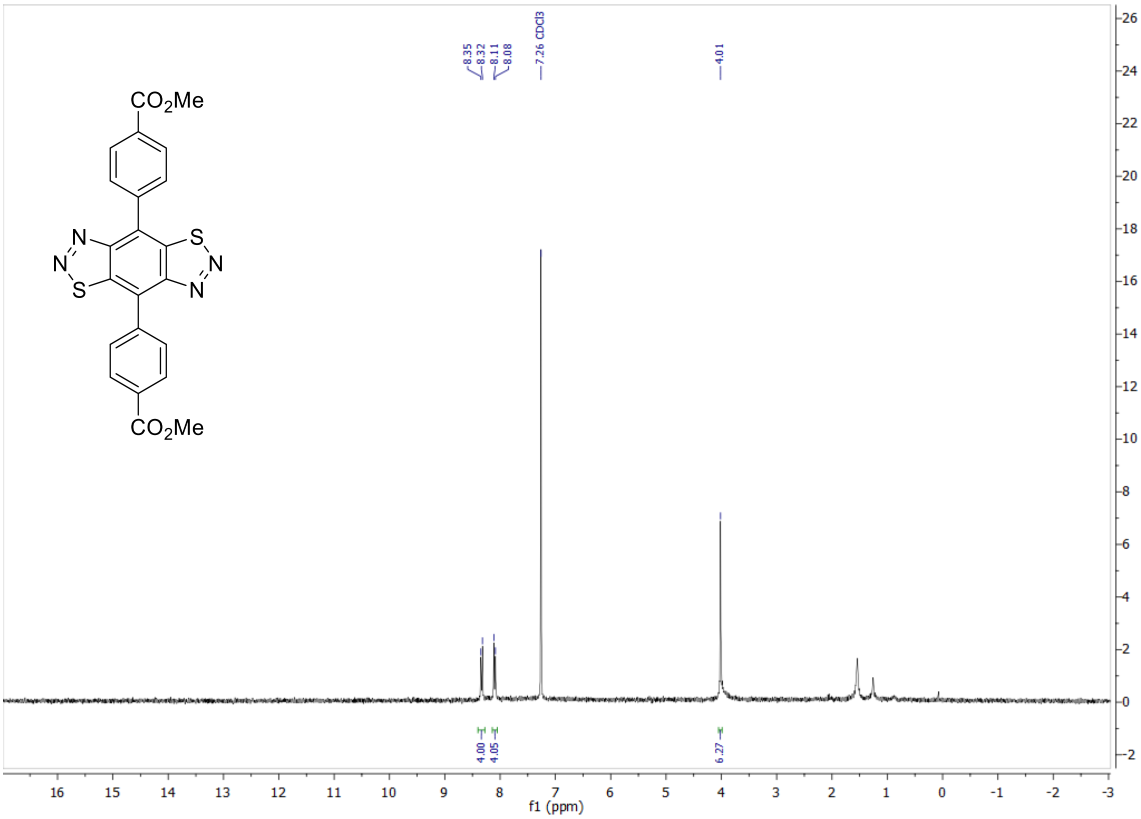

^{13}C NMR(75 MHz)

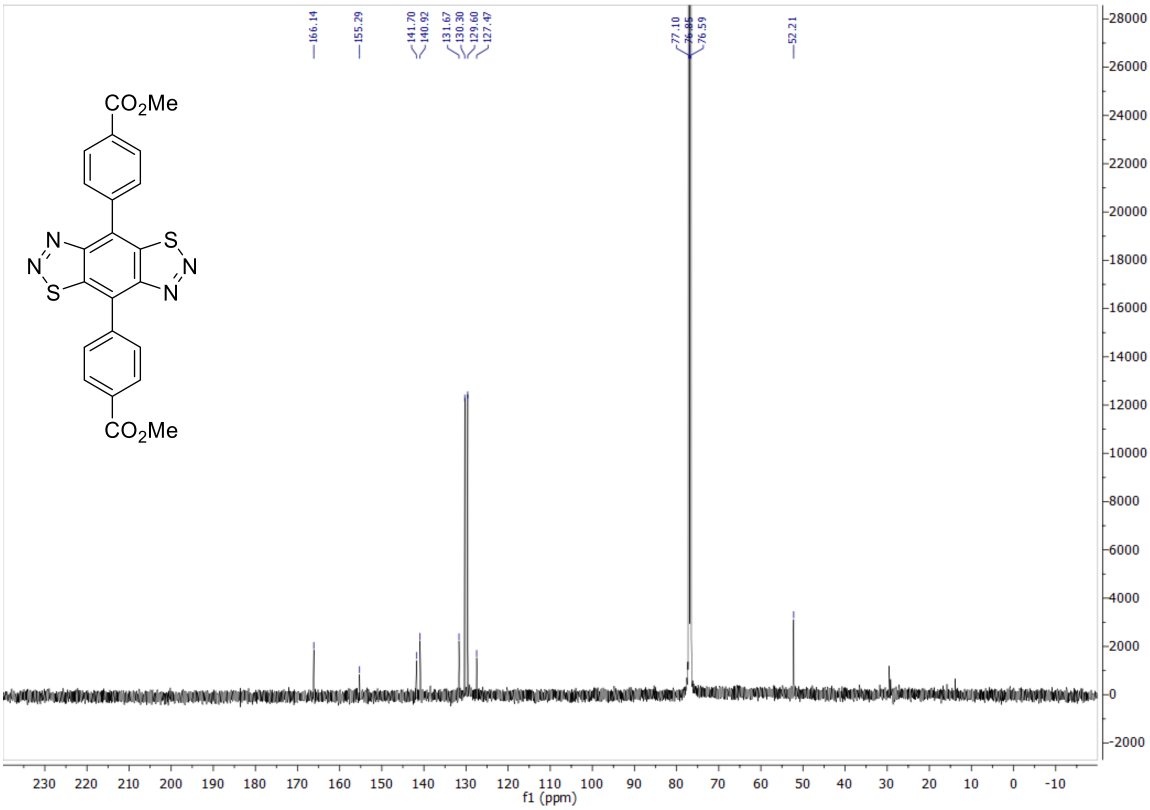

4,4'-(Benzo[1,2-d:4,5-d']bis([1,2,3]thiadiazole)-4,8-diyl)bis(N,N-diphenylaniline) (5j)

^{1}H NMR (300 MHz)

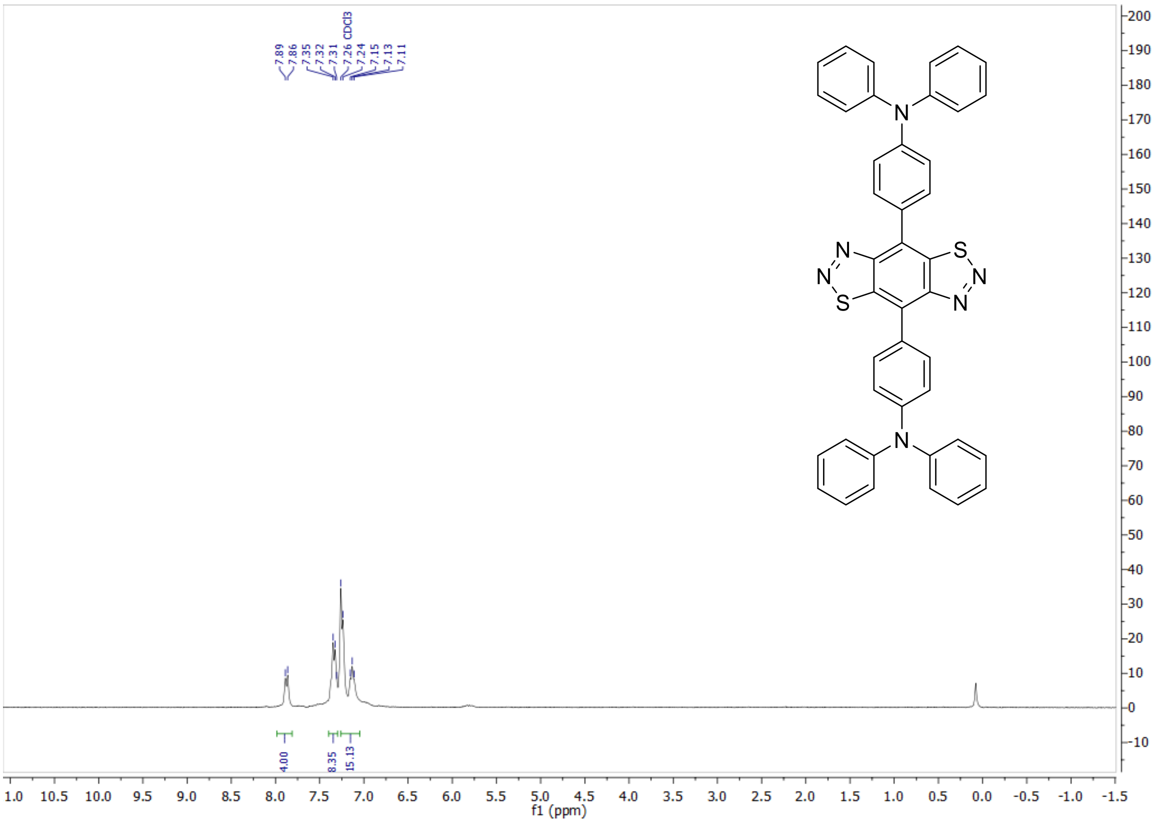

^{13}C NMR(75 MHz)

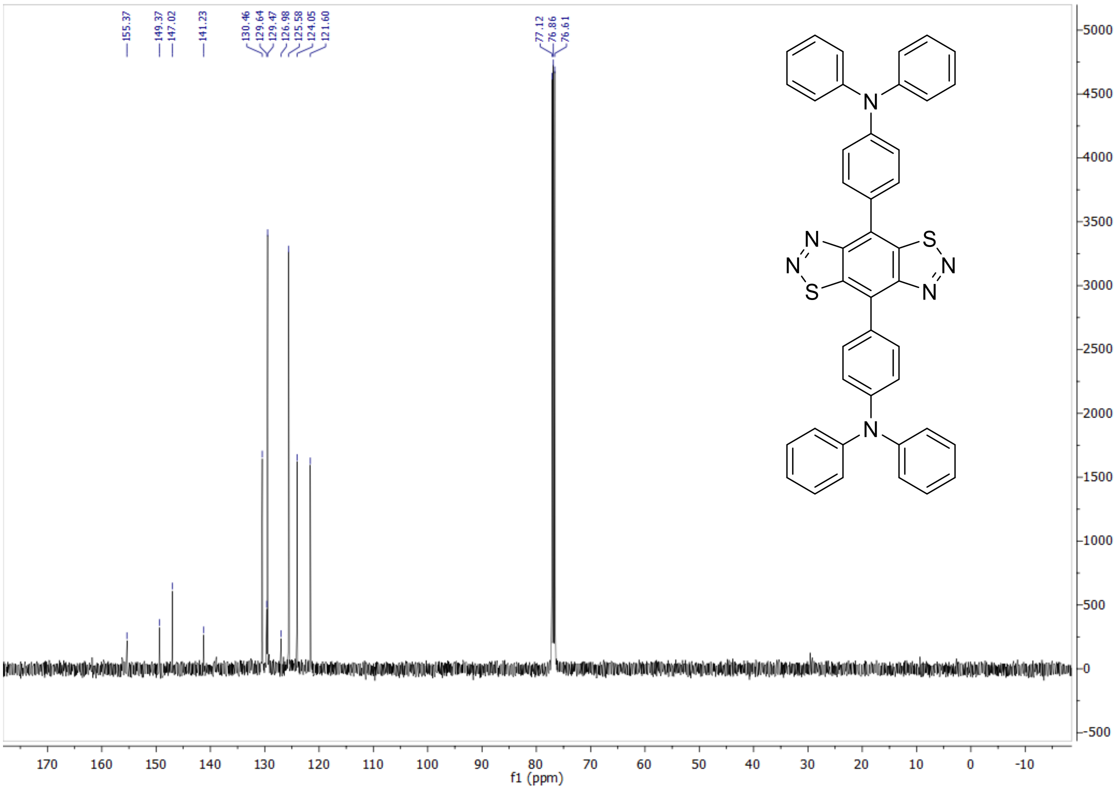

4-(5-(2-Ethylhexyl)thiophen-2-yl)benzo[1,2-*d*:4,5-*d'*]bis([1,2,3]thiadiazole) (7a)

^{1}H NMR (300 MHz)

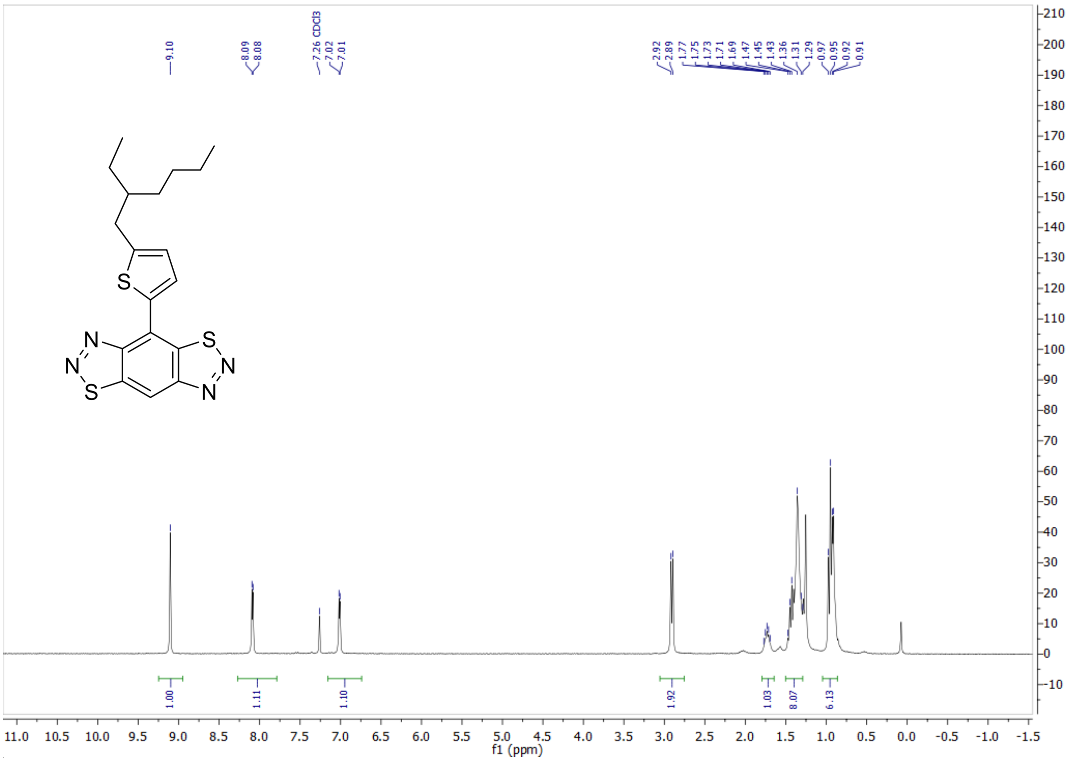

^{13}C NMR(75 MHz)

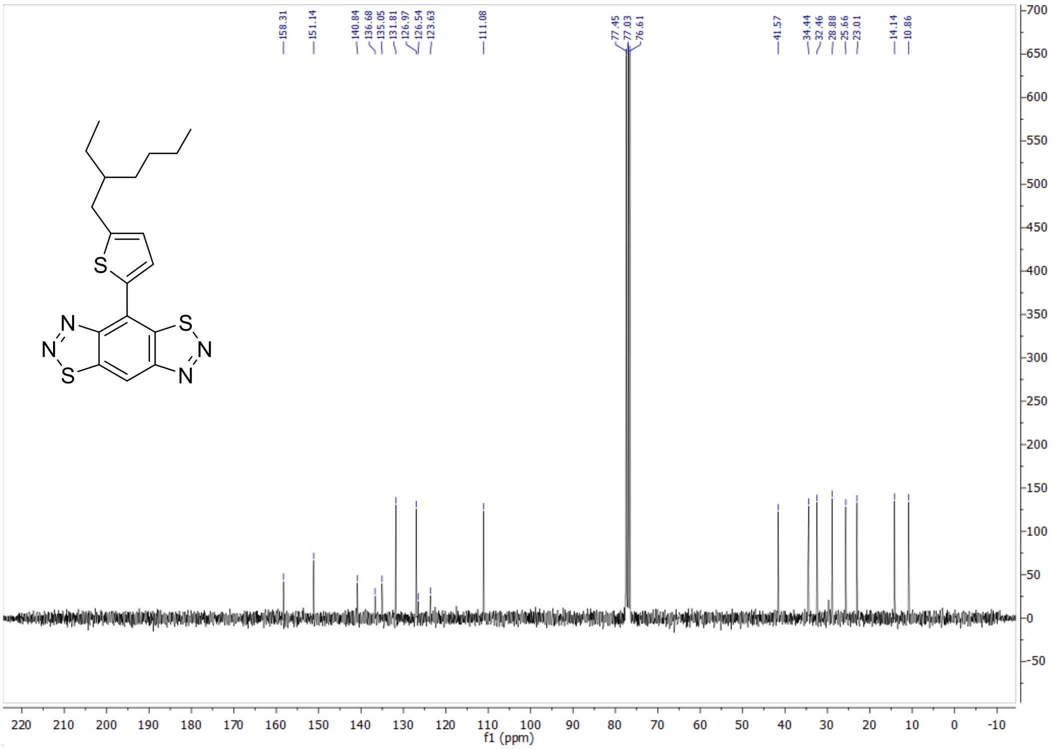

4-(Thiophen-2-yl)benzo[1,2-d:4,5-d']bis([1,2,3]thiadiazole) (7b)

^{1}H NMR (300 MHz)

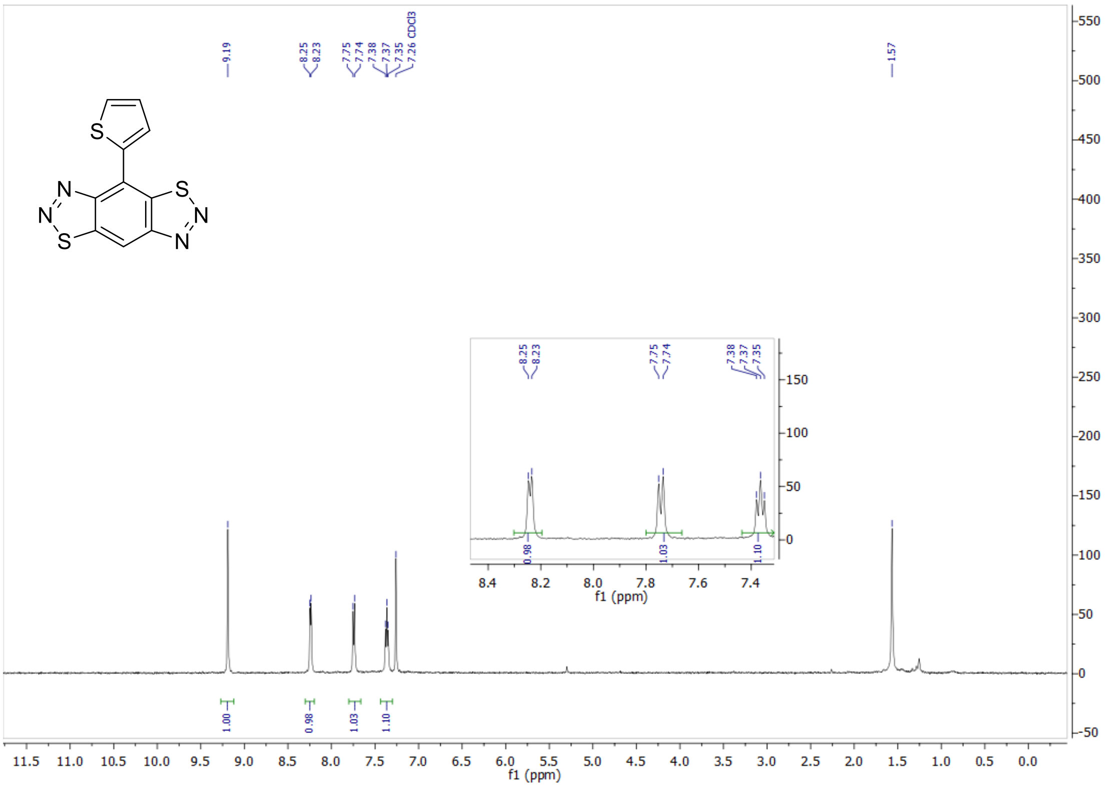

^{13}C NMR(75 MHz)

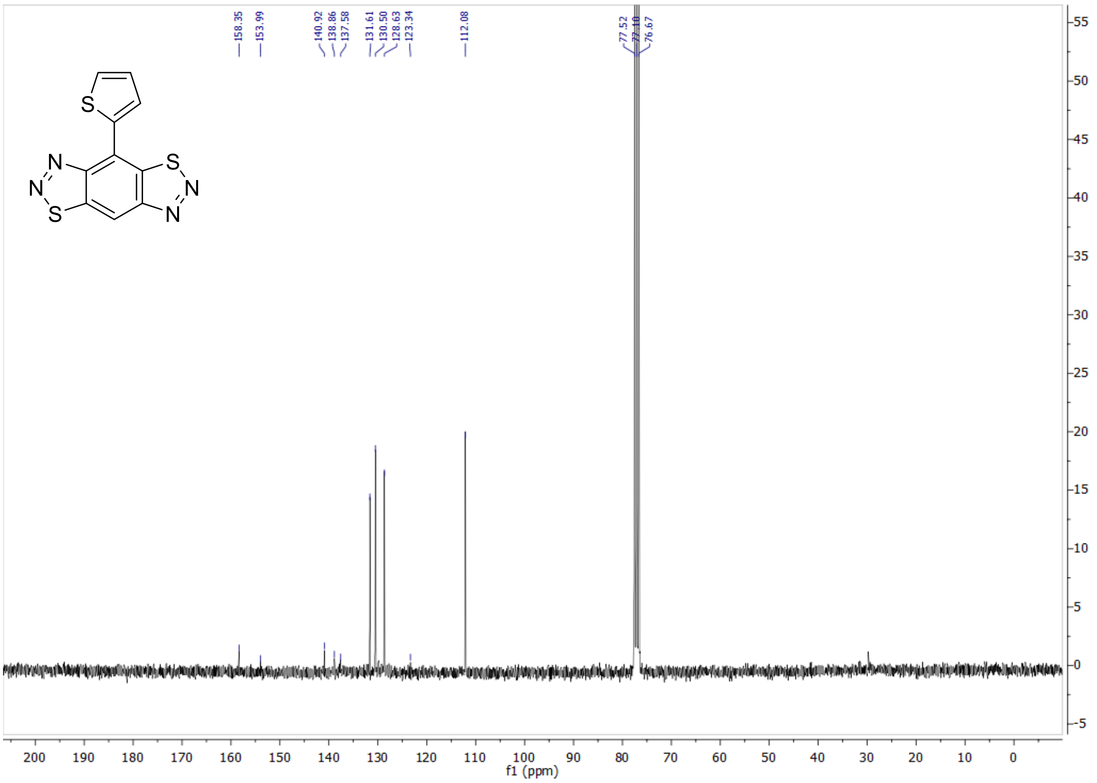

4-(4-Hexylthiophen-2-yl)benzo[1,2-d:4,5-d']bis([1,2,3]thiadiazole) (7c)

^{1}H NMR (300 MHz)

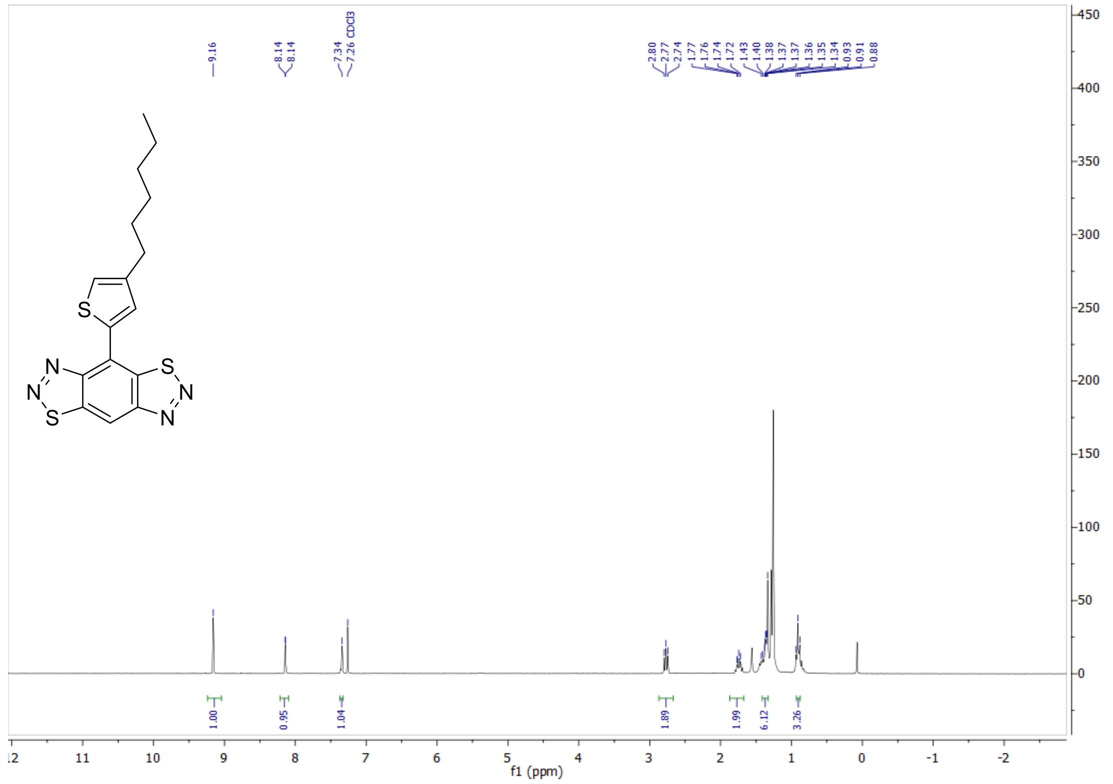

^{13}C NMR(75 MHz)

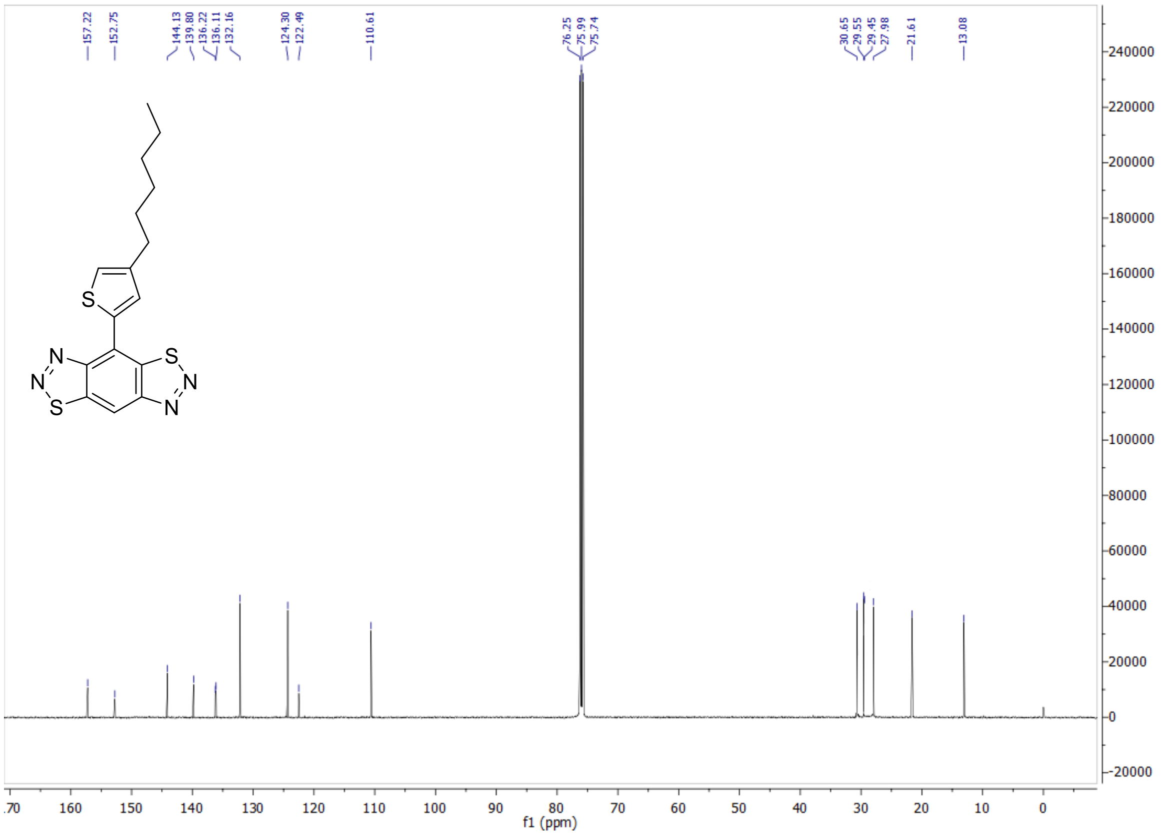

4-(5'-(Trimethylsilyl)-[2,2'-bithiophen]-5-yl)benzo[1,2-*d*:4,5-*d*']bis([1,2,3]thiadiazole) (7e)

^{1}H NMR (300 MHz)

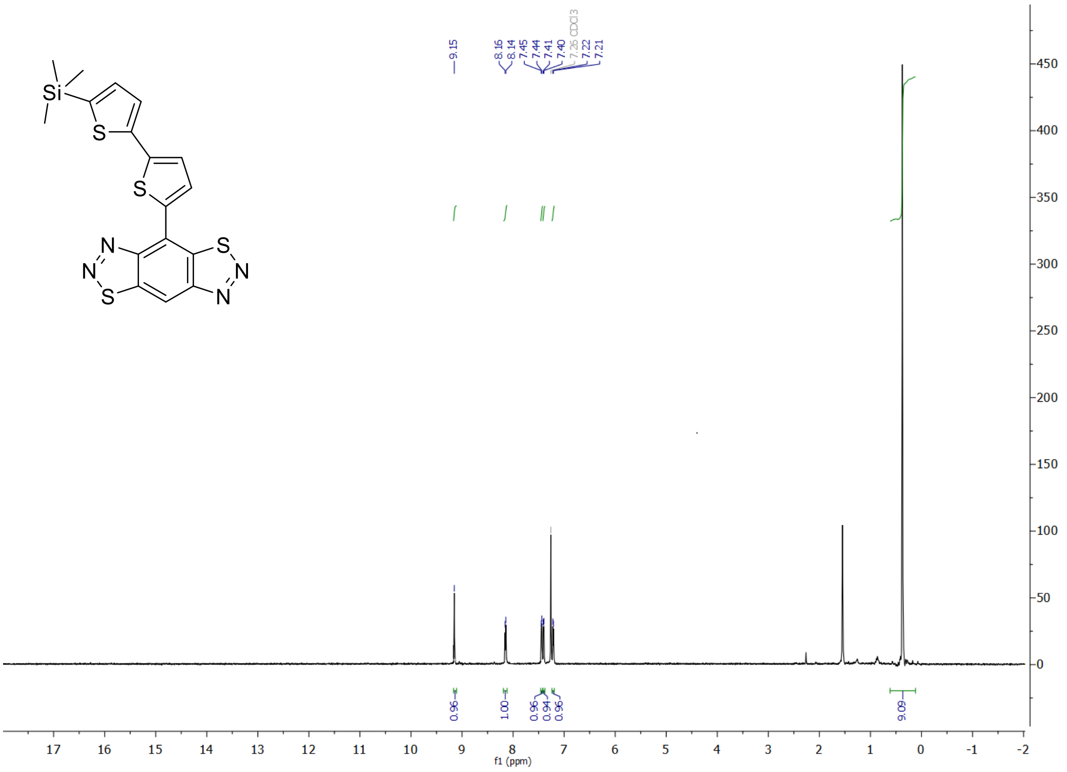

^{13}C NMR(75 MHz)

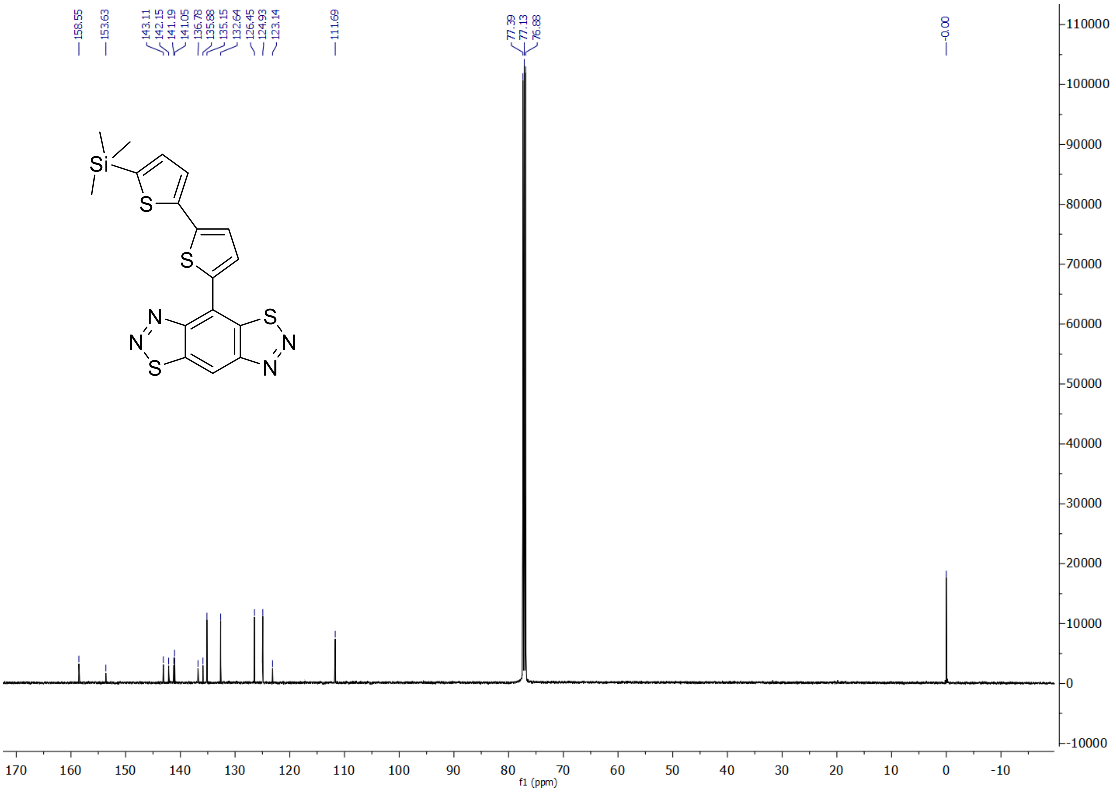

4-Phenylbenzo[1,2-*d*:4,5-*d'*]bis([1,2,3]thiadiazole) (7f)

^{1}H NMR (300 MHz)

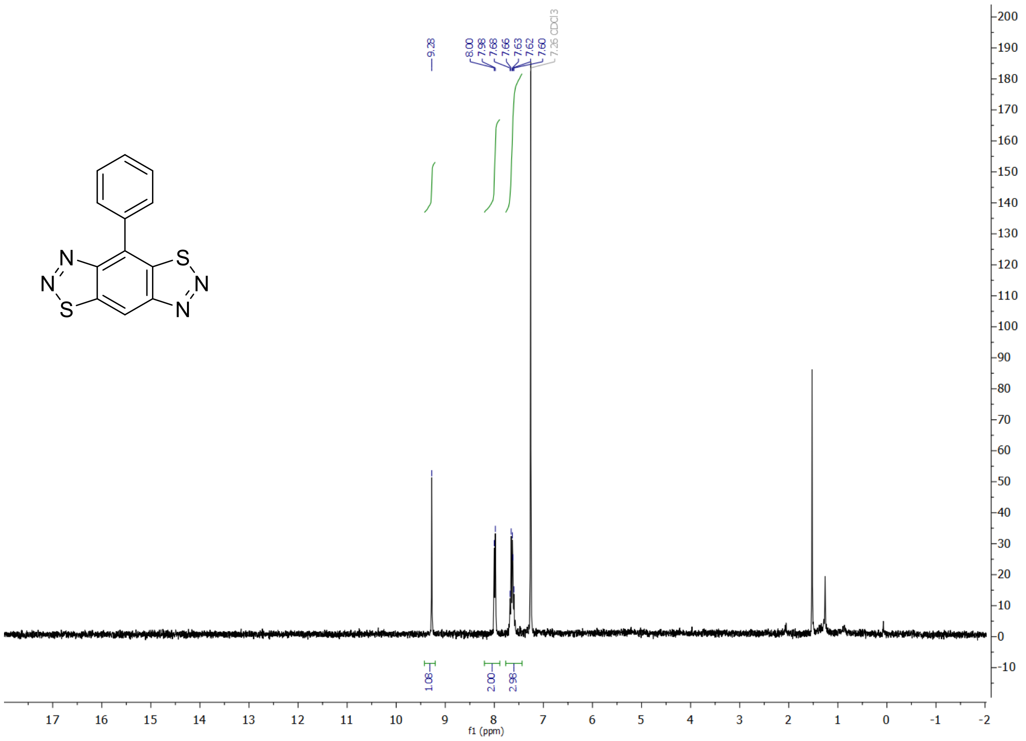

^{13}C NMR(75 MHz)

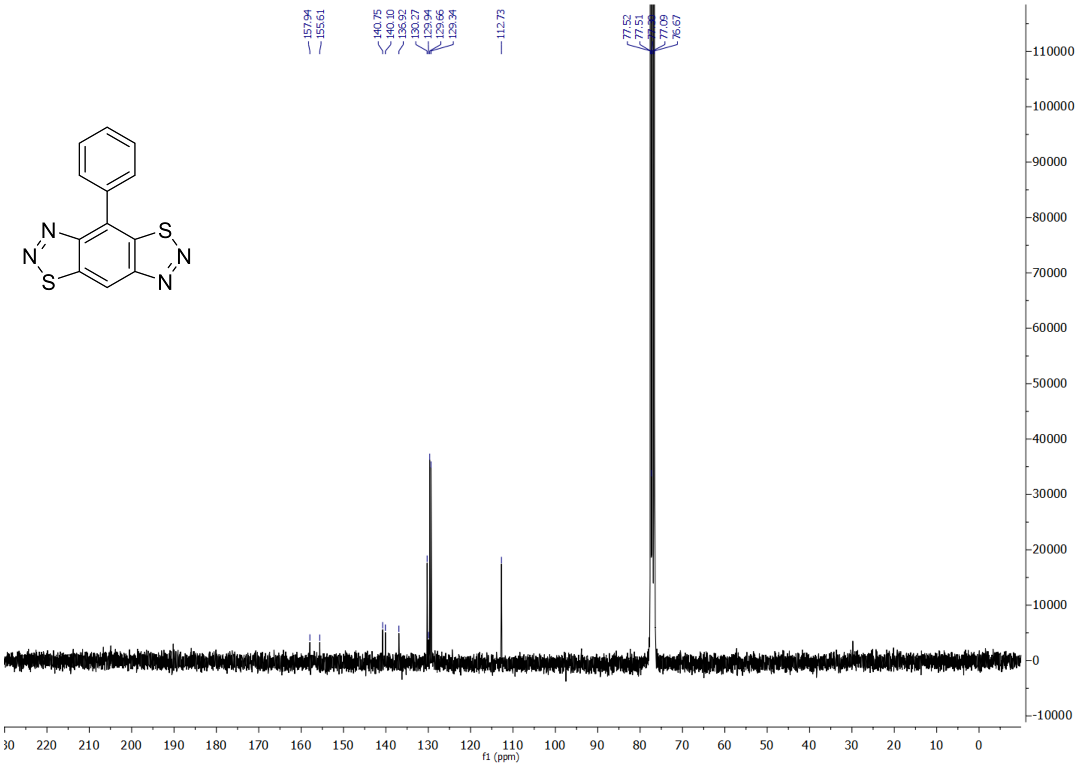

4-(p-Tolyl)benzo[1,2-d:4,5-d']bis([1,2,3]thiadiazole) (7g)

^{1}H NMR (300 MHz)

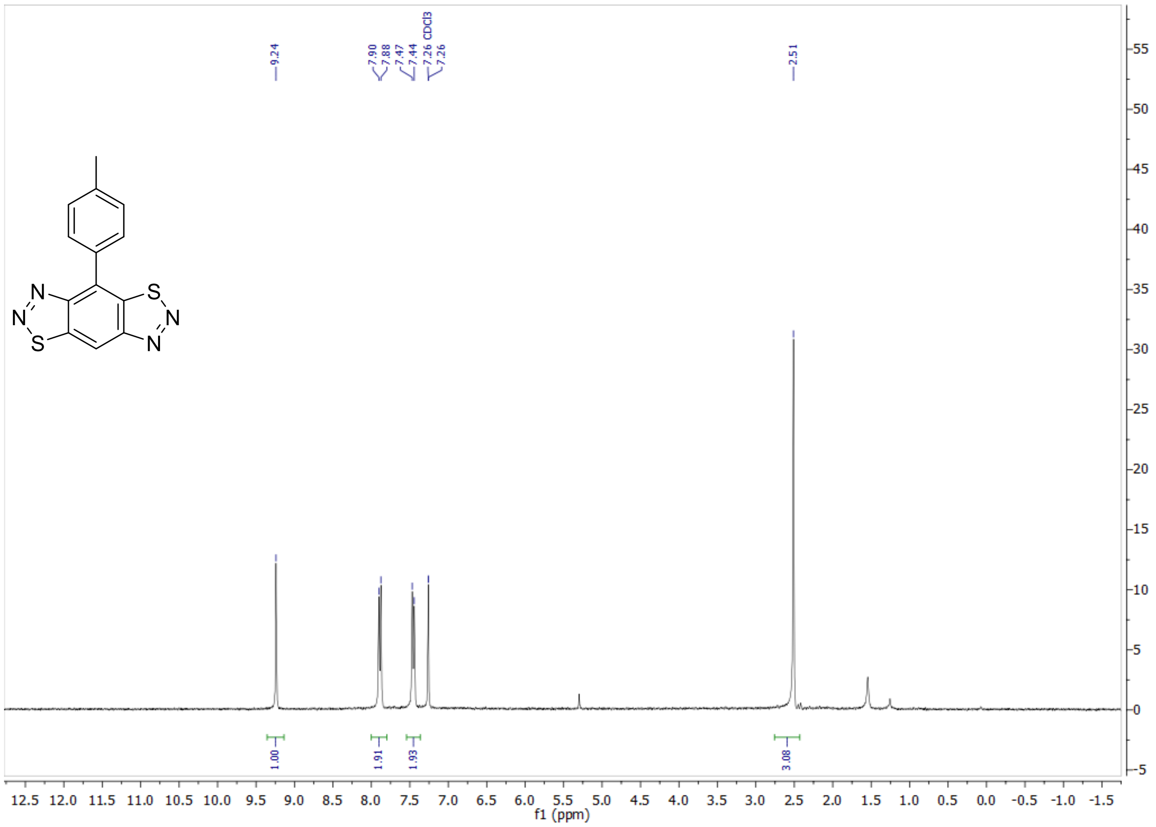

^{13}C NMR(75 MHz)

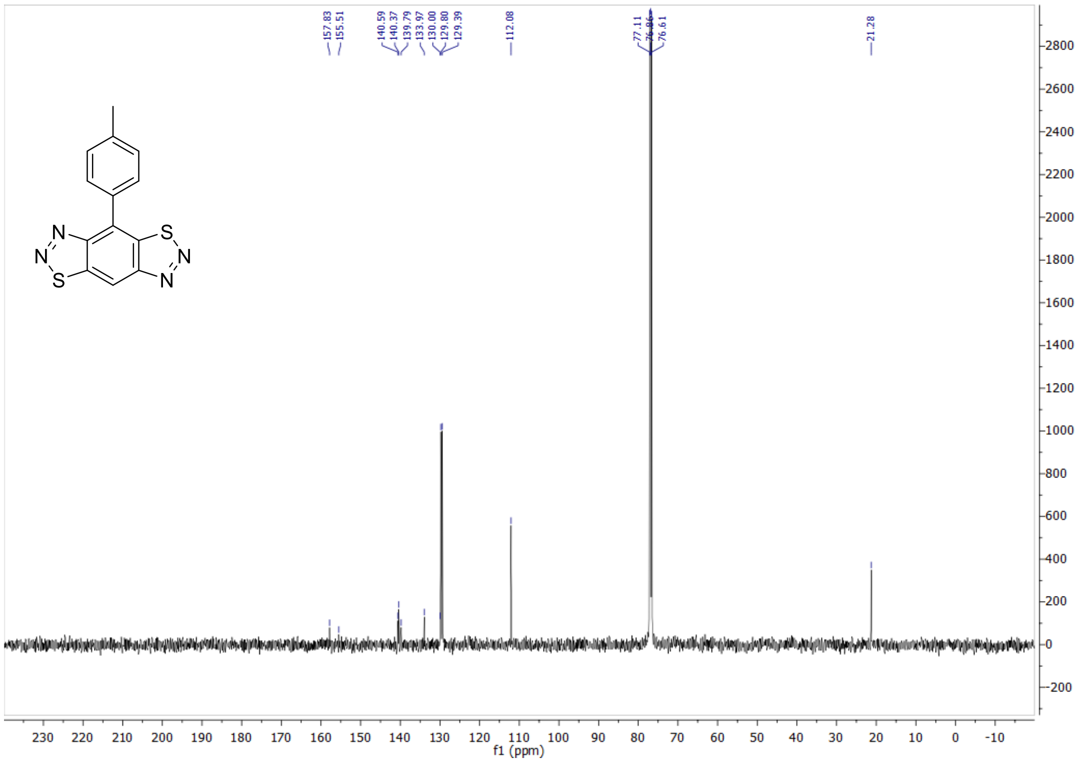

4-(4-Methoxyphenyl)benzo[1,2-*d*:4,5-*d*']bis([1,2,3]thiadiazole) (7h)

^{1}H NMR (300 MHz)

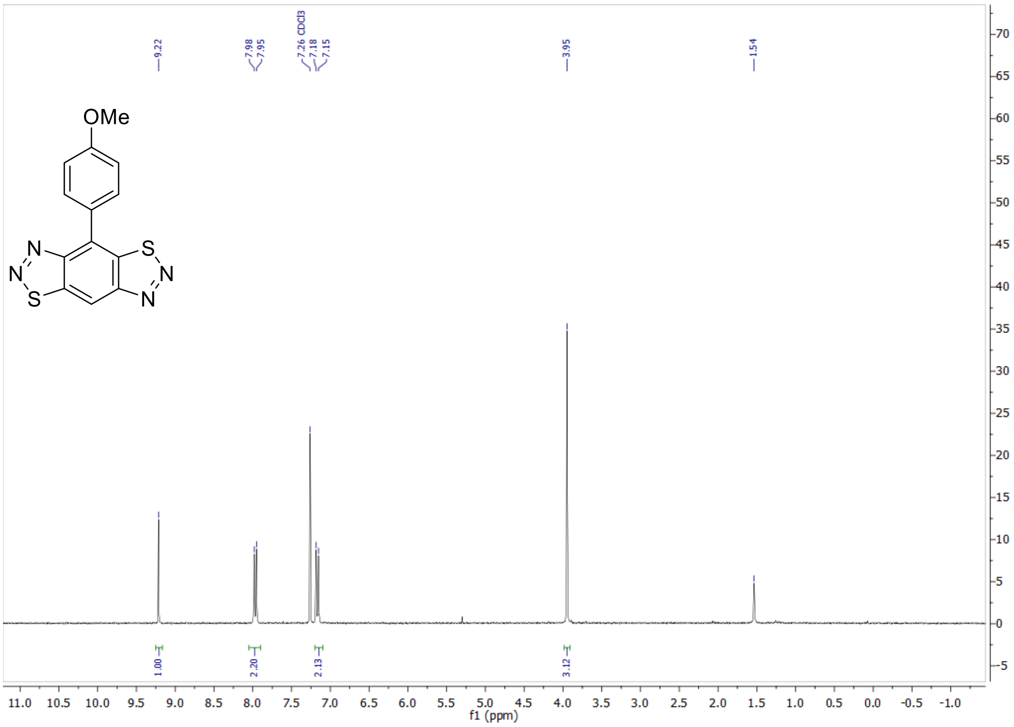

^{13}C NMR(75 MHz)

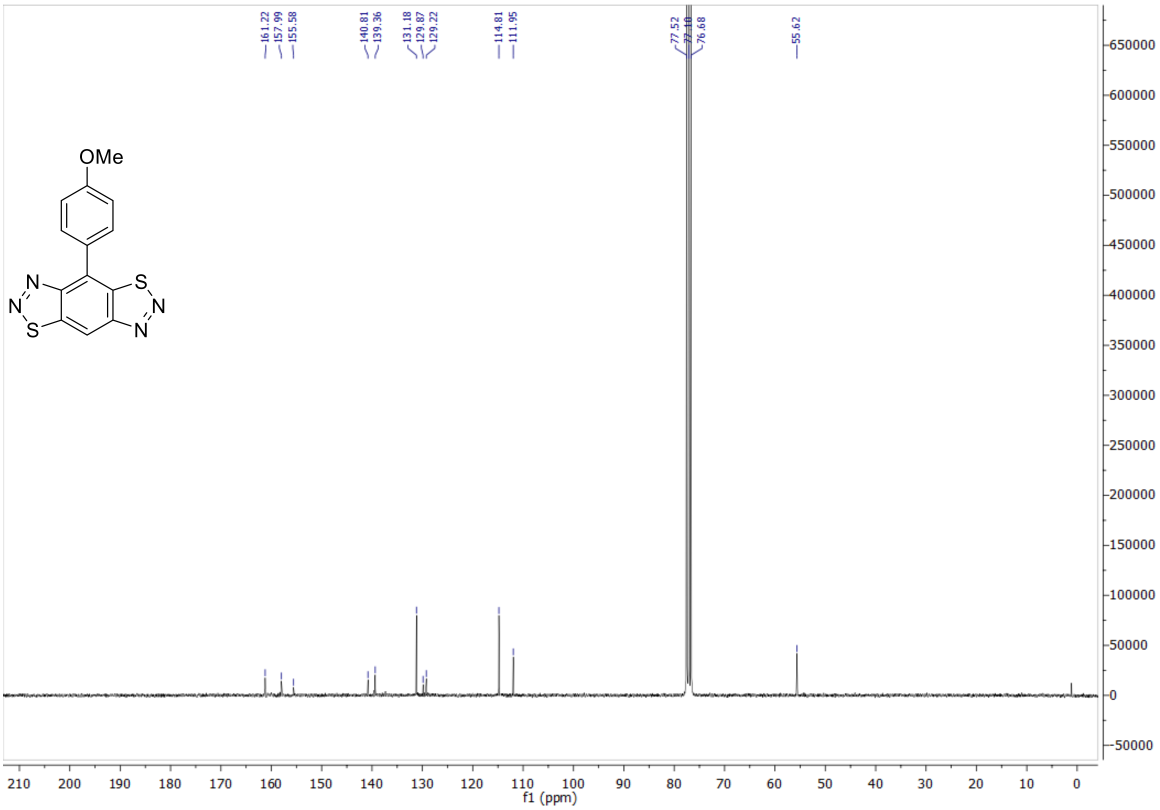

Methyl 4-(benzo[1,2-*d*:4,5-*d'*]bis([1,2,3]thiadiazole)-4-yl)benzoate (7i)

^{1}H NMR (300 MHz)

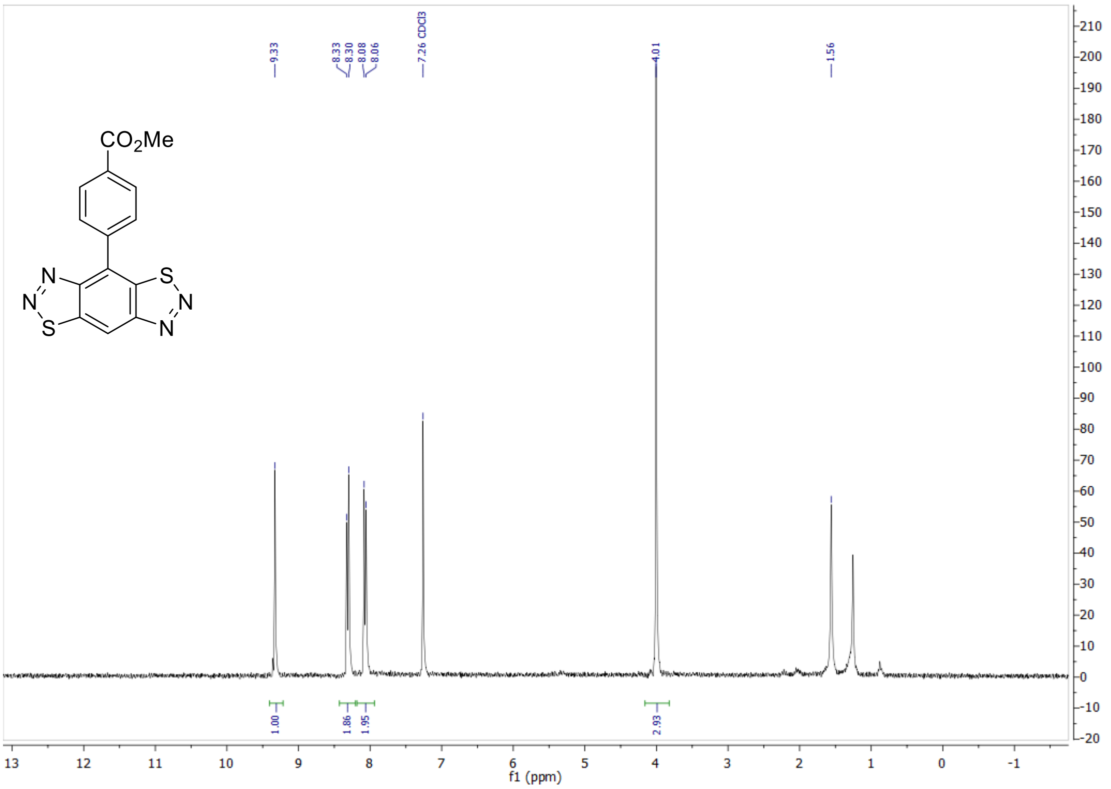

^{13}C NMR(75 MHz)

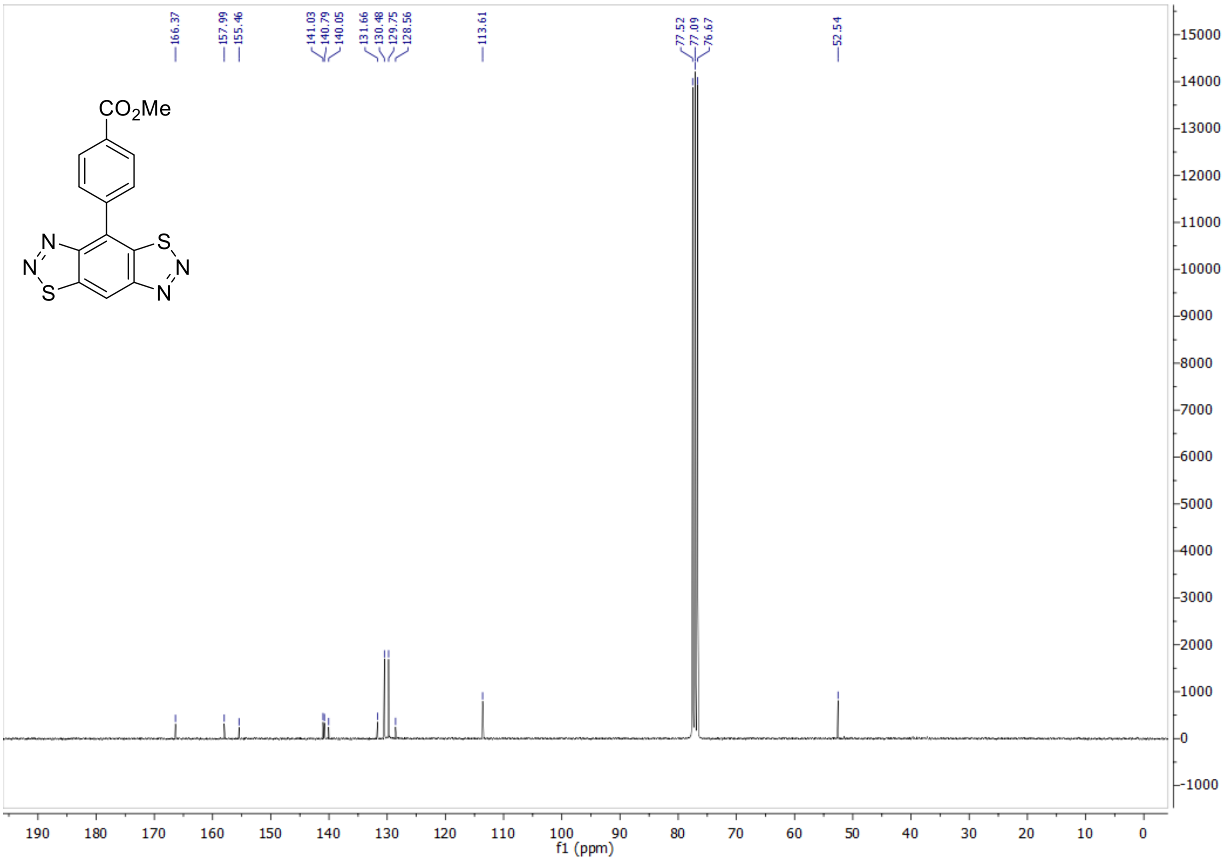

4-(Benzo[1,2-*d*:4,5-*d'*]bis([1,2,3]thiadiazole)-4-yl)-*N*,*N*-diphenylaniline (7j)

^{1}H NMR (300 MHz)

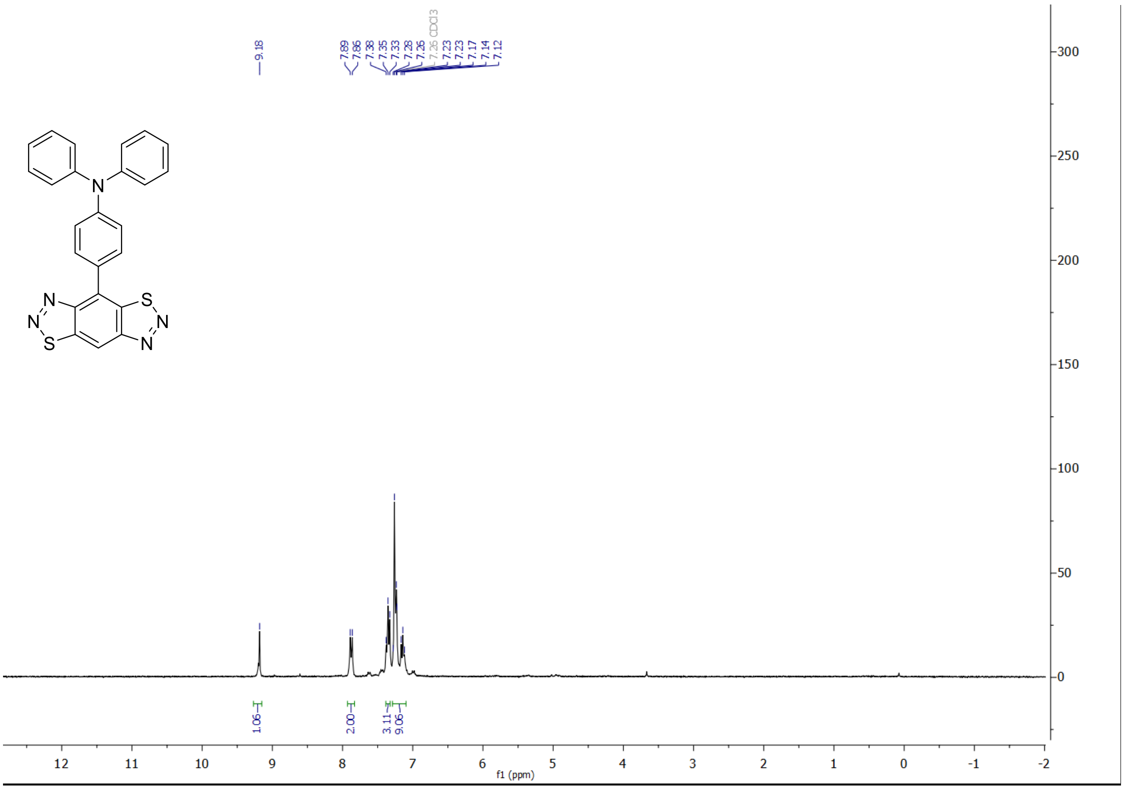

^{13}C NMR(75 MHz)

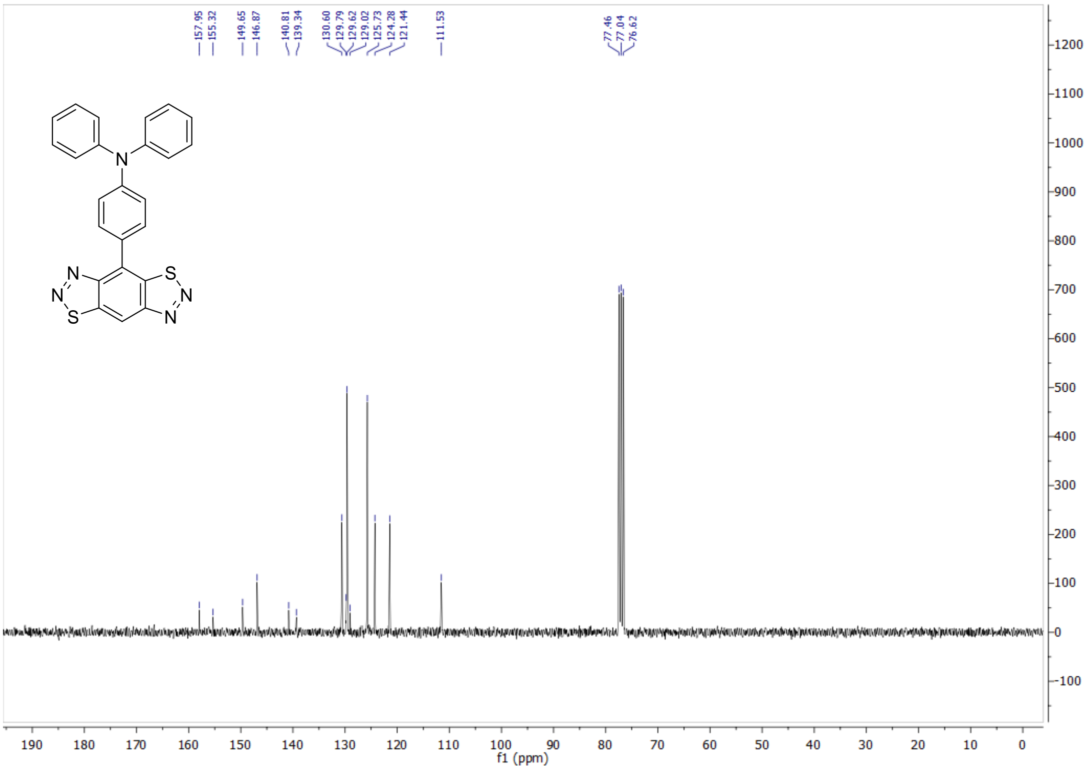

Supplement: Supplementary file 1 [file molecules-28-03977-s001.zip › molecules-2373824-supplementary.pdf]
